# Overcome Chemoresistance: Biophysical and Structural Analysis of Synthetic FHIT-Derived Peptides

**DOI:** 10.3389/fmolb.2021.715263

**Published:** 2021-11-25

**Authors:** Maria Carmina Scala, Simone Di Micco, Delia Lanzillotta, Simona Musella, Veronica Di Sarno, Barbara Parrino, Stella Maria Cascioferro, Giuseppe Bifulco, Francesco Trapasso, Pietro Campiglia, Marina Sala

**Affiliations:** ^1^ Department of Pharmacy, University of Salerno, Fisciano, Italy; ^2^ European Biomedical Research Institute of Salerno (EBRIS), Salerno, Italy; ^3^ Department of Experimental and Clinical Medicine, University Magna Græcia, Campus S. Venuta, Catanzaro, Italy; ^4^ Department of Biological Chemical and Pharmaceutical Sciences and Technologies (STEBICEF), University of Palermo, Palermo, Italy

**Keywords:** chemoresistance, peptide, FHIT, annexin A4, biophysical assay

## Abstract

The fragile histidine triad (FHIT) protein is a member of the large and ubiquitous histidine triad (HIT) family of proteins. On the basis of genetic evidence, it has been postulated that the FHIT protein may function as tumor suppressor, implying a role for the FHIT protein in carcinogenesis. Recently, Gaudio et al. reported that FHIT binds and delocalizes annexin A4 (ANXA4) from plasma membrane to cytosol in paclitaxel-resistant lung cancer cells, thus restoring their chemosensitivity to the drug. They also identified the smallest protein sequence of the FHIT still interacting with ANXA4, ranging from position 7 to 13: QHLIKPS. This short sequence of FHIT protein was not only able to bind ANXA4 but also to hold its target in the cytosol during paclitaxel treatment, thus avoiding ANXA4 translocation to the inner side of the cell membrane. Starting from these results, to obtain much information about structure requirements involved in the interaction of the peptide mentioned above, we synthetized a panel of seven peptides through an Ala-scan approach. In detail, to study the binding of FHIT derived peptides with ANXA4, we applied a combination of different biophysical techniques such as differential scanning fluorimetry (DSF), surface plasmon resonance (SPR), and microscale thermophoresis (MST). Circular dichroism (CD) and nuclear magnetic resonance (NMR) were used to determine the conformational structure of the lead peptide (7–13) and peptides generated from ala-scan technique. The application of different biophysical and structural techniques, integrated by a preliminary biological evaluation, allowed us to build a solid structure activity relationship on the synthesized peptides.

## Introduction

Chemoresistance is the ability of cancer cells to evade the action of several classes of anti-cancer drugs, thus becoming a serious problem that cancer research seeks to understand and overcome.

The molecular mechanisms of how cancer cells promote their own survival and avoid apoptosis in response to commonly used chemotherapeutics are multiple and consist of a set of signaling pathways, which can be activated by a large amount of stimuli to promote chemoresistance (Housman et al., 2014; Bukowski et al., 2020). In this regard, ANXA4 is an interesting “unconventional” oncoprotein being its overexpression is involved in chemoresistance (Mogami et al., 2013). ANXA4 belongs to the family of annexins that are proteins able to bind calcium ions and phospholipids and contribute to biological processes such as endocytosis, exocytosis, cell division, apoptosis, and growth regulation (Gerke et al., 2005; Mussunoor and Murray, 2008).

The involvement of ANXA4 in chemoresistance, partly based on cellular efflux mediated by the copper transporter ATP7A, and its overexpression in solid tumors has been extensively discussed (Matsuzaki et al., 2014; Morimoto et al., 2014).

Recently, Gaudio et al. reported a chemoresistance model after paclitaxel treatment. They demonstrated that paclitaxel administration induces both ANXA4 up-regulation and modification of its intracellular distribution; in fact, following paclitaxel treatment, ANXA4 moves from cytosol to cell membrane. Moreover, they observed in *Fhit*-negative lung cancer cells that the overexpression of FHIT protein, a molecule lost early in the majority of human tumors, avoided this subcellular relocalization thus restoring the sensitivity of cancer cells to paclitaxel-induced apoptosis, both *in vitro* and *in vivo* (Gaudio et al., 2013). In fact, FHIT protein is a member of the HIT family of proteins that may function as tumor suppressor (Brenner, 2002; Kiss et al., 2017). Afterward Gaudio et al. also identified the smallest sequence of the FHIT protein still interacting with ANXA4. This short sequence, QHLIKPS, ranging from position 7 to 13 of FHIT protein, was not only able to bind ANXA4 but also to hold it in the cytosol during paclitaxel treatment, thus avoiding ANXA4 translocation to the inner side of cell membrane (Gaudio et al., 2016).

Considering FHIT mimetic peptide 7–13 (peptide **1**, Table 1) as a candidate leading molecule for the development of therapeutic chemical compounds targeting ANXA4, in this work, we initiated a systematic structure−activity relationship (SAR) study in order to identify the crucial residues for the interaction with ANXA4. The development of direct binding assays allowed to evaluate the affinity of the peptides derived from FHIT 7–13 with ANXA4. Conformational studies by CD and NMR were realized to deep investigate the structure of synthesized peptides. Biological assays were performed to explore the ability of peptides to influence the cell viability in lung cancer cells. Among the different peptides designed, one peptide was identified for its capability to bind ANXA4 and reduce cell viability in paclitaxel-treated lung cancer cells.

**TABLE 1:** The binding affinities between ANXA4 and peptide 1−8 determined using MST and SPR.

Peptide	Sequence	K_D_ (μM) MST	K_D_ (μM) SPR
**1**	QHLIKPS	4.65 ± 0.02	0.84 ± 0.05
**2**	QHLIKPA	0.218 ± 0.016	0.552 ± 0.08
**3**	QHLIKAS	0.014 ± 0.003	0.375 ± 0.02
**4**	QHLIAPS	0.146 ± 0.013	0.053 ± 0.01
**5**	QHLAKPS	1.41 ± 0.35	NPD
**6**	QHAIKPS	6.4 ± 0.26	NPD
**7**	QALIKPS	1.13 ± 0.05	0.85 ± 0.055
**8**	AHLIKPS	3.04 ± 0.080	1.52 ± 0.33

NPD, nonpertinent data

## Materials and Methods

### Chemicals

N-α-Fmoc-protected amino acids, coupling reagents 1-Hydroxy-7-azabenzotriazole (HOAt) and 2-(1H-benzotriazole-1-yl)-1,1,3,3-tetramethyluronium hexafluoro-phosphate (HBTU), N, N-Diisopropylethylamine (DIEA), piperidine and trifluoroacetic acid (TFA) were purchased from Iris Biotech (Germany). Rink Amide-ChemMatrix resin was purchased from Biotage AB (Sweden). Peptide synthesis solvents, reagents, as well as CH_3_CN for High Performance Liquid Chromatography (HPLC) were reagent grade and were acquired from commercial sources and used without further purification unless otherwise noted. Ultrapure water (H_2_O) was obtained by a Direct-8 Milli-Q system (Millipore, Milan, Italy).

### Microwave Peptide Synthesis

The synthesis of FHIT derivatives (**1–8**) was performed according to the solid phase approach using standard Fmoc methodology by Biotage Initiator + Alstra automated microwave synthesizer.

Peptides were synthesized on a Rink Amide-ChemMatrix resin (150 mg, loading 0.47 mmol/g), previously swollen in Dichloromethane (DCM, 1 × 3 min, 1 × 10 min) at room temperature (rt).

The resin was then washed with N, N-dimethylformamide (DMF, 4 × 4.5 ml) and the first protected amino acid as well as the following one were added on to the resin stepwise. Coupling reactions were achieved using N-α-Fmoc amino acids (4.0 eq., 0.5 M), HBTU (3 eq, 0.6 M), HOAt (3 eq, 0.5 M), and DIEA (6 eq, 2 M) in N-methyl-2-pyrrolidone (NMP) for 10 min at 75°C (2×) and 2 × 45 min at room temperature (rt) for histidine couplings to avoid the epimerization.

After each coupling step, the Fmoc protecting group was removed with 30% piperidine/DMF (1 × 3 min, 1 × 10 min) at rt. The resin was washed with DMF (4 × 4.5 ml) after each coupling and deprotection step. The N-terminal Fmoc group was removed as described above, and the peptides were acetylated adding a solution of Ac_2_O/DCM (1:3) shaking for 30 min. Finally, the peptides were released from the resin with TFA/iPr_3_SiH/H_2_O (90:5:5) for 3 h. The resin was removed by filtration and the crude peptide recovered by precipitation with cold anhydrous ethyl ether to give a white powder that was then lyophilized.

### Purification and Characterization

Crude peptides were purified by RP-HPLC on a preparative C18-bonded silica column (Phenomenex Kinetex AXIA 100 Å, 100 × 21.2 mm, 5 µm) using a Shimadzu SPD 20 A UV/VIS detector, with detection at 220 and 254 nm. The column was perfused at a flow rate of 17 ml/min with solvent A (5%, v/v, water in 0.1% aqueous TFA) and a linear gradient from 5 to 90% of solvent B (85%, v/v, acetonitrile in 0.1% aqueous TFA) over 20 min was adopted for peptide elution. Analytical purity and retention time (t_r_) of each peptide were determined using HPLC conditions in the above solvent system (solvents A and B) at a flow rate of 0.800 ml/min using a linear gradient from 5 to 90% B over 11 min, fitted with C-18 column Supelco, Ascentis express peptide C18 column (50 × 3.00 mm, 2.7 µm). All analogues showed >97% purity when monitored at 220 nm. Homogeneous fractions, as established using analytical HPLC, were pooled and lyophilized. Peptides molecular weights were determined by positive ESI infusion on a Q-Tof Premier Mass Spectrometer (Waters), equipped with the Xcalibur software for processing the data acquired. The sample was dissolved in a mixture of water and methanol (50/50) and injected directly into the electrospray source, using a syringe pump, at constant flow (15 μL/min). Analytical data are shown in the supplementary material.

### Binding Measurement Assays

His-tag-ANXA4 was expressed by Genscript (lot N. U8886ED090-5/P3ED001). CM5 sensor chips, HBS-P+ buffer (0.01 M HEPES pH 7.4, 0.15 M NaCl, 0.05% v/v Surfactant P20), 1-ethyl-3-(3-diaminopropyl) carbodiimide hydrochloride (EDC), N-hydroxysuccinimide (NHS), ethanolamine (H_2_N(CH_2_)_2_OH), and regeneration solution were purchased from Cytiva.

#### Microscale Thermophoresis

Microscale Thermophoresis experiments were performed on a Monolith NT.115pico (NanoTemper Techonologies, Munich, Germany). ANXA4 was labelled with His-Tag Labeling Kit RED-tris-NTA (Nanotemper, cat n° NT-L118). Briefly, 100 μL of an 80 nM solution of ANXA4 protein in labeling buffer (Phosphate buffered saline (PBS), pH 7.4) was mixed with 100 μL of 40 nM NT647-NHS fluorophore (NanoTemper Technologies) in labeling buffer and incubated for 30 min at rt. For storage, NT647-ANXA4 was frozen in 10 μL aliquots at −80°C prior to MST experiments; the aliquots of NT647-ANXA4 were thawed on ice and centrifuged for 10 min at 4°C at 15,000 g to remove protein aggregates.

Pretests using standard-treated and premium-coated MST capillaries (NanoTemper Technologies) were performed to test for adsorption of ANXA4 to capillary walls by analyzing capillary scans recorded by the Monolith NT.115pico prior to MST experiments. The protein did not adsorb to standard-treated capillary walls in assay buffer (PBS, pH 7.4, 5 mM CaCl_2_). For this reason, the following experiments were performed using standard-treated capillary. Then, buffer conditions were evaluated to identify optimal state for MST signal reproducibility and the suppression of unspecific adsorption to capillary walls. Compound stocks (1 mM) in PBS were serially diluted in assay buffer to reach the highest soluble concentration (50 µM). Each dilution was mixed 1:1 with a solution of 40 nM NT647-ANXA4 to yield a final volume of 20 μL. After 10 min of incubation at rt, the reaction mixtures were loaded into standard-treated capillaries and subsequently analyzed by high MST power and a light-emitting diode intensity of 10%.

K_D_ values were calculated from compound concentration-dependent changes in normalized fluorescence (F_norm_) of ANXA4 after 20 s of thermophoresis. Each compound was tested in duplicate samples, and data were analyzed using MO Affinity Analysis software (NanoTemper Technologies).

#### Surface Plasmon Resonance

The affinity of synthetic peptides for ANXA4 was determined by SPR using a Biacore T200 (GE Health Sciences Inc.). His-tag-ANXA4 were stably captured at the surface of the CM5 sensor chip by means of an anti-histidine antibody (His Capture Kit, GE Healthcare) that had been covalently bound to the surface as recommended by the manufacturer. In particular, the anti-histidine antibody provided in His Capture Kit was diluted to 50 μg/ml in the immobilization buffer included in the kit and covalently coupled to Sensor Chip CM5 by standard amine coupling to a level of approximately 12,000 RU. Then, His-tag-ANXA4 was injected (8 μg mL^−1^ in 10 mm acetate, pH 4.5) over the anti-histidine antibody surface for 420s at a flow rate of 5 μl/min. No protein was injected over the reference surface. The dissociation was monitored by injecting running buffer for 600 s. Surface regeneration was done by injecting glycine buffer (10 mM, pH 1.5, 1 min).

HBS-P+ buffer (pH 7.4 with 5 mM CaCl_2_) was used as a running buffer. After the immobilization, assay buffer was injected over the chip at a flow rate of 5 μL/min overnight. A solution of peptide in HBS-P + buffer at various concentrations (from 0.08 to 5 µM) was injected at 25°C with a flow rate of 30 μL/min for 120 s (association phase), and then the buffer alone was injected for 600 s (dissociation phase).

The equilibrium dissociation constants (K_D_) and kinetic dissociation (kd) and association (ka) constants were calculated from the sensorgrams by global fitting of a 1:1 binding model using analysis software (v2.02) provided with the Biacore T200 instrument (GE Healthcare).

#### Nanoscale Differential Scanning Fluorometry

The assay was carried out in PBS (pH 7.4, 5 mM CaCl_2_) buffer using 40 μM of protein and 200 μM of compounds. The samples were loaded into nanoDSF Grade Standard Capillaries (NanoTemper Technologies) and analyzed using the Prometheus NT.48 nanoDSF device (NanoTemper Technologies). Thermal unfolding of the protein was monitored using a linear thermal ramp (1°C/min; 20°C–95°C) with an excitation power of 90%. Binding affinity of peptides was estimated from the ratio of 330 and 350 nm estimated at 20°C and 95°C for the native and unfolded forms of the protein, respectively. In control, the sample was replaced with buffer solution. Three independent measurements were carried out for each compound, and their mean is depicted. The fluorescence intensity ratio and its first derivative were calculated with the manufacturer’s software (PR.ThermControl, version 2.1.2).

### Circular Dichroism

All CD spectra were recorded using a JASCO J810 spectropolarimeter at 25°C between *λ* = 260–190 nm (1 mm path, 1 nm bandwidth, 4 accumulations, and a scanning speed of 10 nm/min). Measurements were performed with peptides dissolved in 10 mM phosphate buffer at pH = 7.4 or in 20 and 40% (HFIP)/PBS solution (HFIP, hexafluoroisopropanol). Estimation of secondary structure content was performed using the algorithms CONTIN from the DICHROWEB website (Whitmore and Wallace, 2004).

### NMR Experiments and Structure Calculation

The NMR samples were obtained dissolving 1 mg of **1** and **3** in hexafluoroacetone:water (10 mM of KH_2_PO_4_) mixture (1:1) and transferred into a 5 mm NMR tube (500 μl). All NMR experiments were carried out at 300 K by Bruker DRX 600 spectrometer equipped with cryoprobe. All spectra were acquired in the phase-sensitive mode, and the TPPI method was used for quadrature detection in the *ω*1 dimension (Marion and Wüthrich, 1983). The residual water signal was suppressed by excitation sculpting with gradients. Data block sizes of 4096 in *t*_2_ and 512 equidistant *t*_1_ values were utilized. The matrices of the time domain data were multiplied by shifted sine bell QSINE (SSB = 2) functions in F1 and F2 dimensions before Fourier transformation. For the 2D-TOCSY experiments a mixing time of 80 ms was applied with 56 scans/*t*1 for **1** and with 70 scans/*t*1 for **3**, whereas for 2D-NOESY experiments mixing times in the range of 300–500 ms were employed with 56 scans/*t*1 for **1** and with 80 scans/*t*1 for **3**. Qualitative and quantitative analyses of 2D spectra were performed by SPARKY software (Goddard and Kneller, 2000). The integrated peak volumes were transformed into upper distance bounds by the CALIBA routine from the CYANA software package (Güntert et al., 1997). The non-stereospecifically assigned protons of methylene and methyl groups were treated by the pseudoatom corrections. The NMR-derived constraints were employed to calculate an ensemble of 200 conformers by the standard CYANA protocol of simulated annealing in the torsion angle space by applying 50,000 steps. The best 20 structures in terms of low target function values and small residual violations, also analyzed by PROCHECK (Laskowski et al., 1996), were selected. All the 3D models were depicted using Maestro 9.6 (Schrodinger, LLC, New York, NY, 2013).

### Biological Assays

#### Cell Culture

A549 were grown in appropriate medium (Sigma Aldrich, St. Louis, MO) supplemented with 10% fetal bovine serum (FBS) (Sigma Aldrich, St. Louis, MO), 1% penicillin/streptomycin (Sigma Aldrich, St. Louis, MO) at 37°C in a 5% CO_2_ incubator.

#### *In vitro* Growth Rate Assessment

There were 500 cells of each cell line (MCF7, PC3, M14, Hela, A549) seeded in 384-mutiwell plates and peptides, dissolved in dimethyl sulfoxide (DMSO), dispensed in each well at different concentrations through Echo Liquid handling system. DMSO were used as a positive control.

A549, adenocarcinoma cells line, were treated using a combo of paclitaxel (800 nM, PTX) and peptides (10 μM, 50 μM, 100 µM). After 24, 48, and 72 h, 10 μL/well of CTG detection mix, from CellTiter-Glo Assay Kit (Promega Corp, Madison, United States), were added; plates were gently mixed, incubated for 10 min in the dark, and read using EnVision Multilabel 2103.

#### Statistics

The statistic performance of each analysis was verified through Z factor (>0.5) and the signal to background (>10).

## Results

### Peptide Design and Synthesis

Recently, the molecular mapping of the Fhit protein domain that interacts with Annexin A4 led to the restriction of the interaction domain to amino acids 7 to 13 of the Fhit protein. This approach, in fact, allowed for the identification of the heptapeptide QHLIKPS (peptide **1**) as the smallest Fhit sequence still able to preserve its ability to bind ANXA4. Fhit peptide also retains the property of the native protein in inhibiting Annexin A4 translocation from cytosol to plasma membrane in A549 and Calu-2 lung cancer cells treated with paclitaxel (Gaudio et al., 2013). In the present study, we performed an L-Ala scanning analysis on peptide **1**, substituting all position-native residues with alanine. This technique allows to determine the contribution of side chains of each amino acid residue in the interaction with the target macromolecule, ANXA4. This approach resulted in the generation of a panel of seven peptides, named **2** to **8** (Table 1).

Peptides were synthesized according to the solid phase approach using standard Fmoc methodology (Carotenuto et al., 2013). The purification was achieved using a semipreparative RP-HPLC C-18 bonded silica column. Pure peptides were characterized by analytical RP-HPLC and mass spectrometry (Materials and Methods).

### Binding Affinity Studies

Biophysical methods are extremely valuable in helping to carry out in depth investigation of protein ligand binding interactions (Holdgate et al., 2019). A correct approach to validate a hit involves the combination of several techniques. As a matter of fact, in this work we performed binding affinity studies by means of three orthogonal techniques: MST, SPR, and DSF.

### Microscale Thermophoresis

**FIGURE 1:**
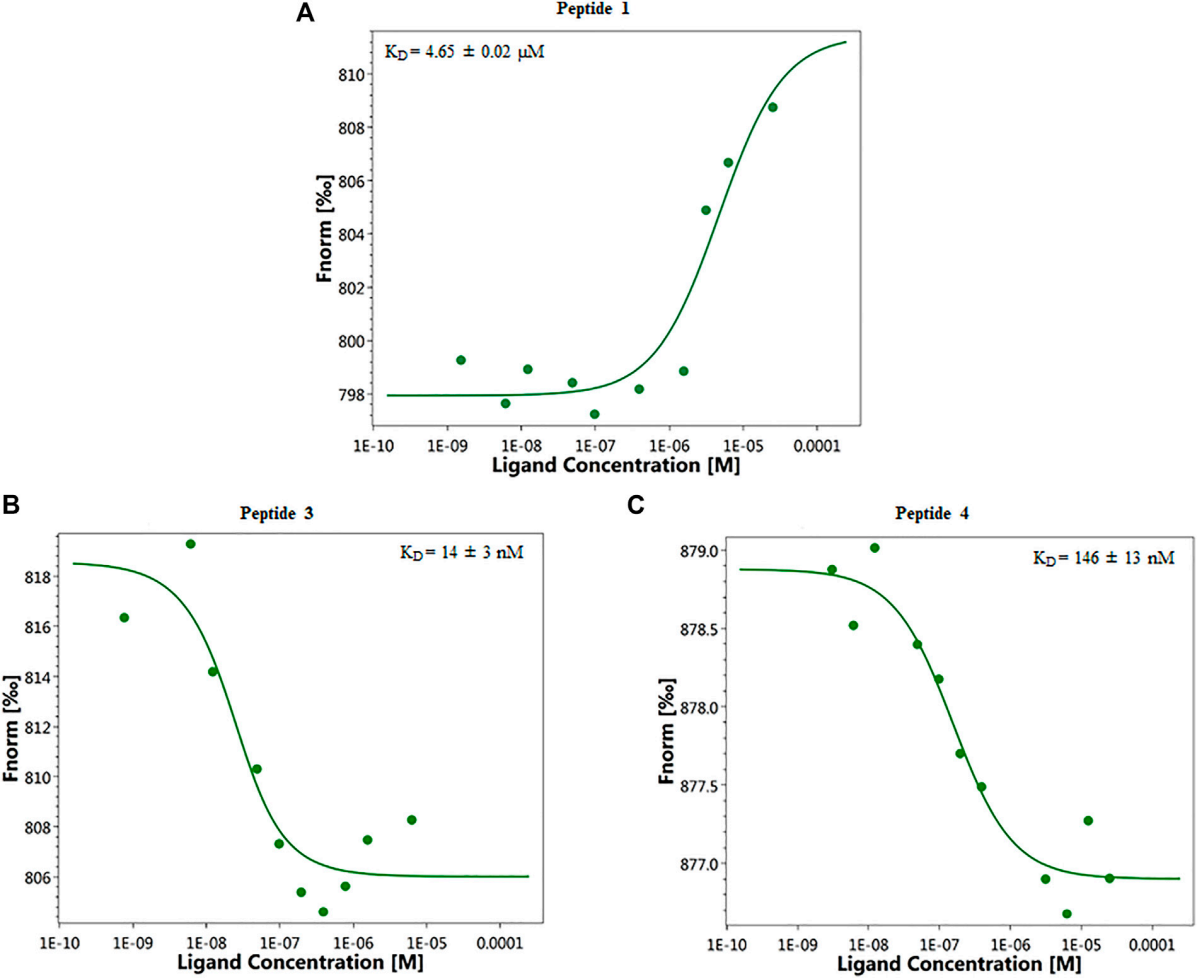
Representative single dose−response curves of **1 (A)**, **3 (B),** and **4 (C)** binding to ANXA4 are shown; F_norm_, normalized fluorescence. Experiments were repeated independently two times. Reported K_D_ is the mean ± SD of two independent experiments.

Binding affinity measurements by MST were realized on a Monolith NT115 system (Nanotemper Technologies, Munich, Germany) for peptides **1**–**8.** The measurement method is based on the directed movement of molecules along a temperature gradient, an effect termed “thermophoresis.” A local temperature difference ΔT leads to a local change in molecule concentration (depletion or enrichment). The K_D_ values are given in Table 1. Results show that peptides **3** and **4** are able to bind ANXA4 with higher efficiency respect to **1**, having dissociation constants of 14 ± 3 nM and 146 ± 13 nM, respectively (Figure 1). The dose-response curves of other compounds are displayed in the Supplementary Material, Supplementary Figures S15–17.

### Surface Plasmon Resonance

SPR assay, also, was performed to evaluate the capability of compounds to bind ANXA4 (Navratilova and Hopkins, 2010). For interaction analyses, the anti-histidine antibody was first covalently immobilized onto the sensor chip CM5. ANXA4 was then injected and captured on the immobilized anti-histidine antibody by means of a 6x-histidine tag fused to the C-terminal part of ANXA4. Then, the analyte sample was injected. A regeneration step was necessary to remove from the sensor chip surface the captured histidine-tagged ligand and any associated molecules (data not shown). After injection, running buffer was allowed to flow over the surface and the dissociation of compounds from the surface was observed. In contrast, the control flow cell, where no ANXA4 was immobilized, showed no significant signal changes (data not shown).

Equilibrium dissociation constant (K_D_) values were derived from the ratio between kinetic dissociation (kd) and association (ka) constants, obtained by fitting data from all injections at different concentrations of each compound using the simple 1:1 Langmuir binding fit model of the BIAevaluation software.

SPR analysis showed the synthetized peptides efficiently interacted with the immobilized protein (Table 1; Supplementary Figures S13–S20). Interestingly, the peptide **3** and **4** bind ANXA4 with higher efficiency with respect to **1**, showing a K_D_ value of 375 ± 20 nM, 53 ± 10 nM, and 0.84 ± 0.05 µM, respectively (Table 1). Larger values were observed for the remaining peptides. In particular, **5**-**8** showed K_D_ similar to **1**.

### Nano Differential Scanning Fluorometry

NanoDSF is an advanced differential scanning fluorimetry method for measuring melting temperature (Tm) of peptide-protein complex. In nanoDSF, a protein in solution is exposed to a temperature gradient that leads to the unfolding of the protein. The intrinsic fluorescence of the protein, mainly originating from the aromatic sidechains of tyrosine and tryptophan residues, is examined. Upon unfolding, the environment of those residues alters because they become exposed to the solvent and, thus, their fluorescence intensity changes. The relation between fluorescence intensity changes and temperature gradient can be used to obtain a so-called apparent Tm. All compounds were screened by nanoDSF at 200 μM concentration and if they exhibited a ΔTm ≥ 0.5°C (chosen as cut-off), they were selected as potential inhibitors. Therefore, compounds **3**-**5** and **7** met the selection criteria exhibiting ΔTm values ranging from 0.5–0.7°C at 200 μM (Table 2).

**TABLE 2:** NanoDSF assays were performed using the Prometheus. The peptides screening was performed at the fixed dose of 200 μM, with a protein concentration of 40 µM. For the analysis, standard capillaries were used.

	Start temperature (°C)	End temperature (°C)	Tm (°C)	ΔTm
**Ctrl**	20	95	48.5	0
**1**	20	95	48.6	0.1
**2**	20	95	48.7	0.2
**3**	20	95	49.0	0.5
**4**	20	95	49.2	0.7
**5**	20	95	49.2	0.7
**6**	20	95	48.9	0.4
**7**	20	95	49.1	0.6
**8**	20	95	48.9	0.4

### Conformational Analysis by Circular Dichroism Studies

**FIGURE 2:**
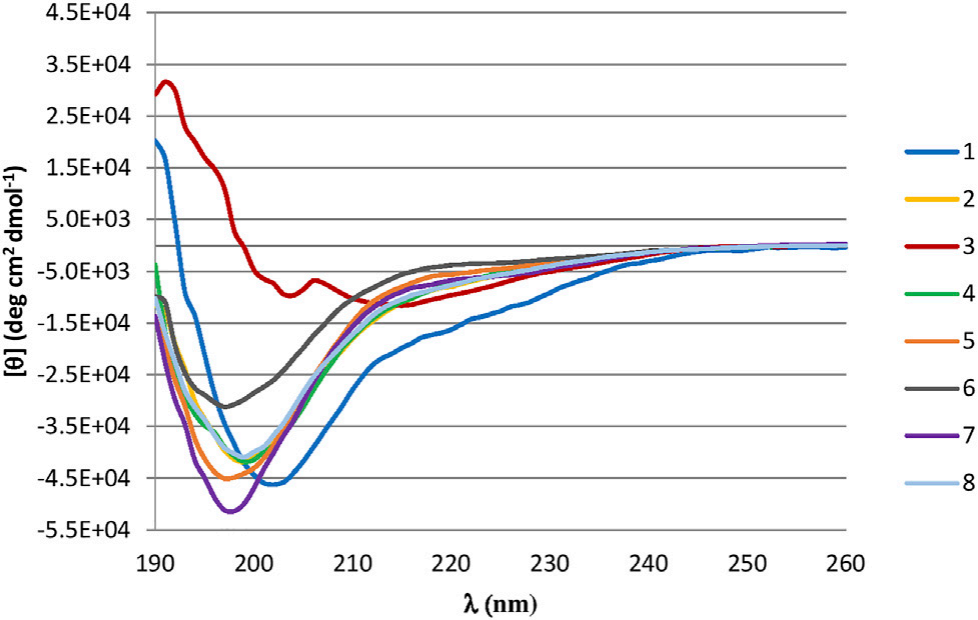
**(A)** CD Spectra of synthesized peptides in 40% HFIP/PBS solution. Peptides are represented in: **1** (blue line), **2** (yellow line), **3** (red line), **4** (green line), **5** (orange line), **6** (grey line), **7** (purple line), **8** (light blue line).

CD was used to determine the conformational structure of the lead peptide (peptide **1**) and peptides generated from ala-scan technique (**2**–**8**). CD studies were performed in PBS, 20 and 40% HFIP/PBS. The peptides showed a random coil conformation in aqueous solution (see Supplementary Material). Spectra acquired in 20% (Figure 2) or 40% HFIP/PBS solution indicate that peptides **2**, **4**–**8** still present an unordered conformation characterized by a single negative band at 198 nm, while the lead peptide **1** adopts a more ordered conformation with a negative band at 200 nM, a weak negative band at 218 nm, and a positive band at 191 nm. Peptide **3** curve presents two negative maxima at 214 nm and at 204 nm and an intensive positive maximum at 192 that is characteristic of the presence of type II β-turn and/or γ-turn (Uray et al., 1999).

### Conformational Analysis by Nuclear Magnetic Resonance Spectroscopy

**FIGURE 3:**
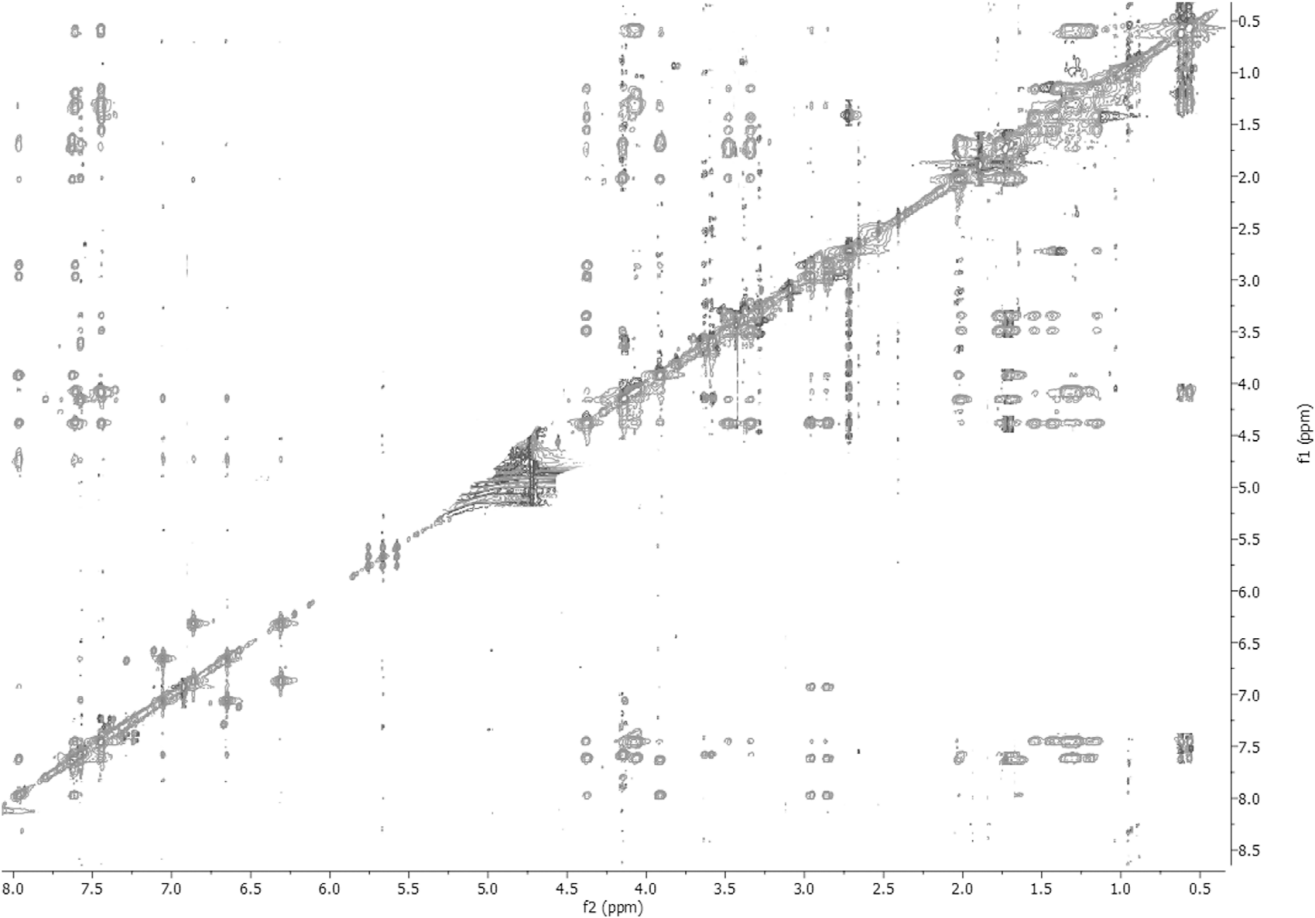
2D-NOESY spectrum of **1** HFA/H_2_O solution (600 MHz, 300 K, *t*_mix_ = 400 ms).

**FIGURE 4:**
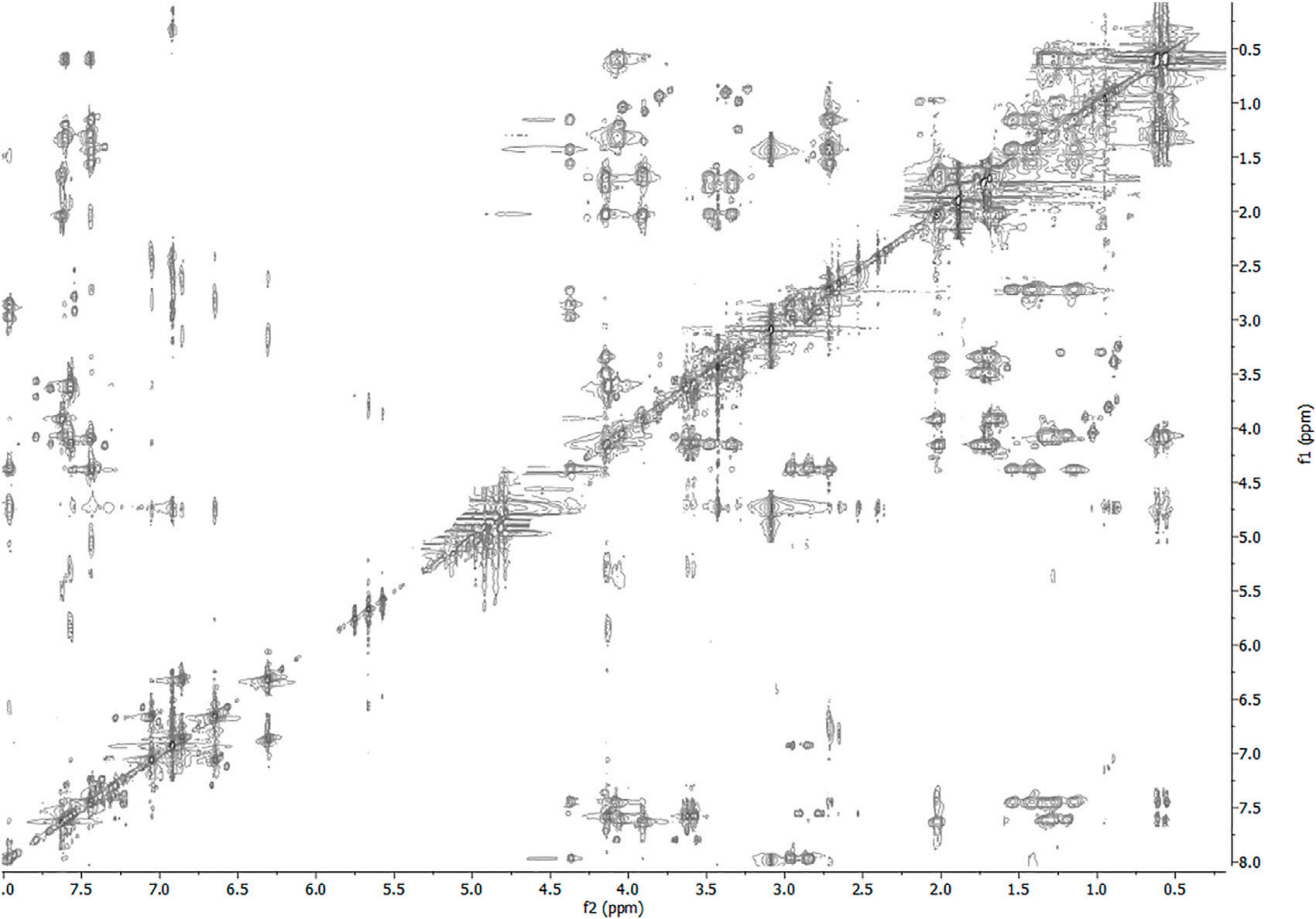
2D-TOCSY spectrum of **1** HFA/H_2_O solution (600 MHz, 300 K, *t*_mix_ = 80 ms).

**FIGURE 5:**
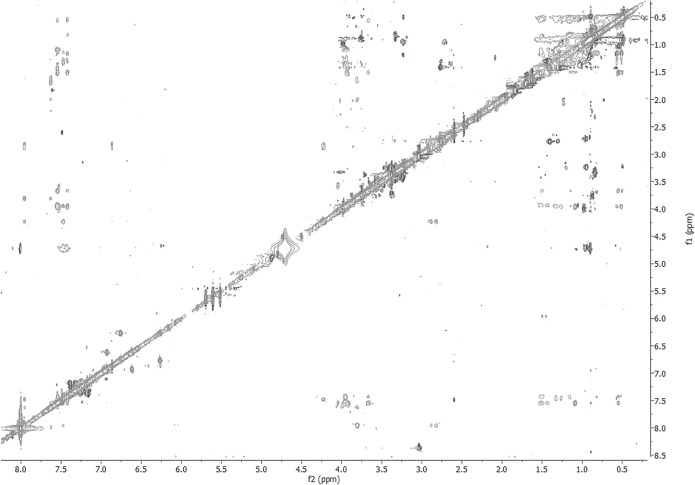
2D-NOESY spectrum of **3** HFA/H_2_O solution (600 MHz, 300 K, *t*_mix_ = 400 ms).

**FIGURE 6:**
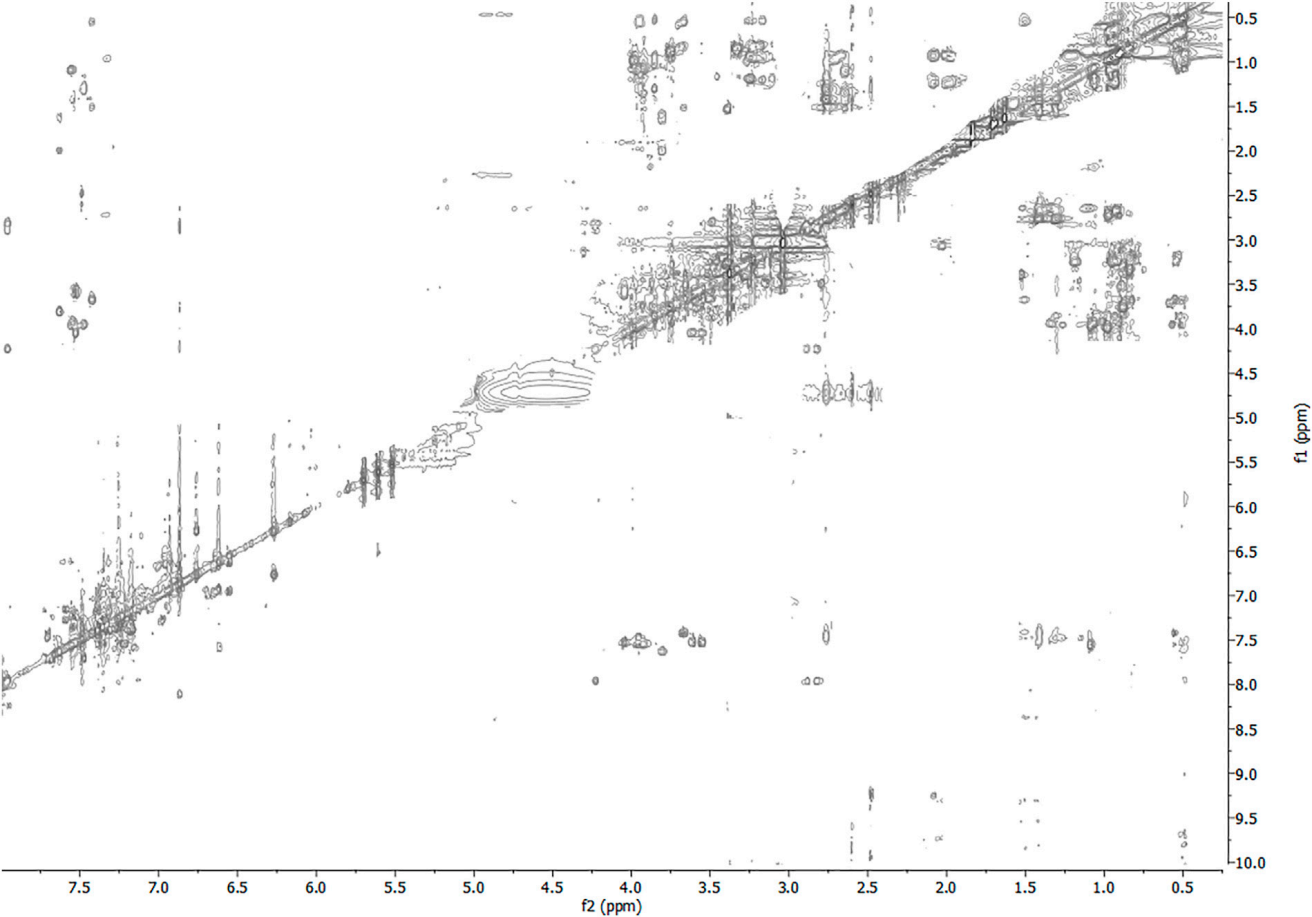
2D-TOCSY spectrum of **3** HFA/H_2_O solution (600 MHz, 300 K, *t*_mix_ = 80 ms).

**FIGURE 7:**
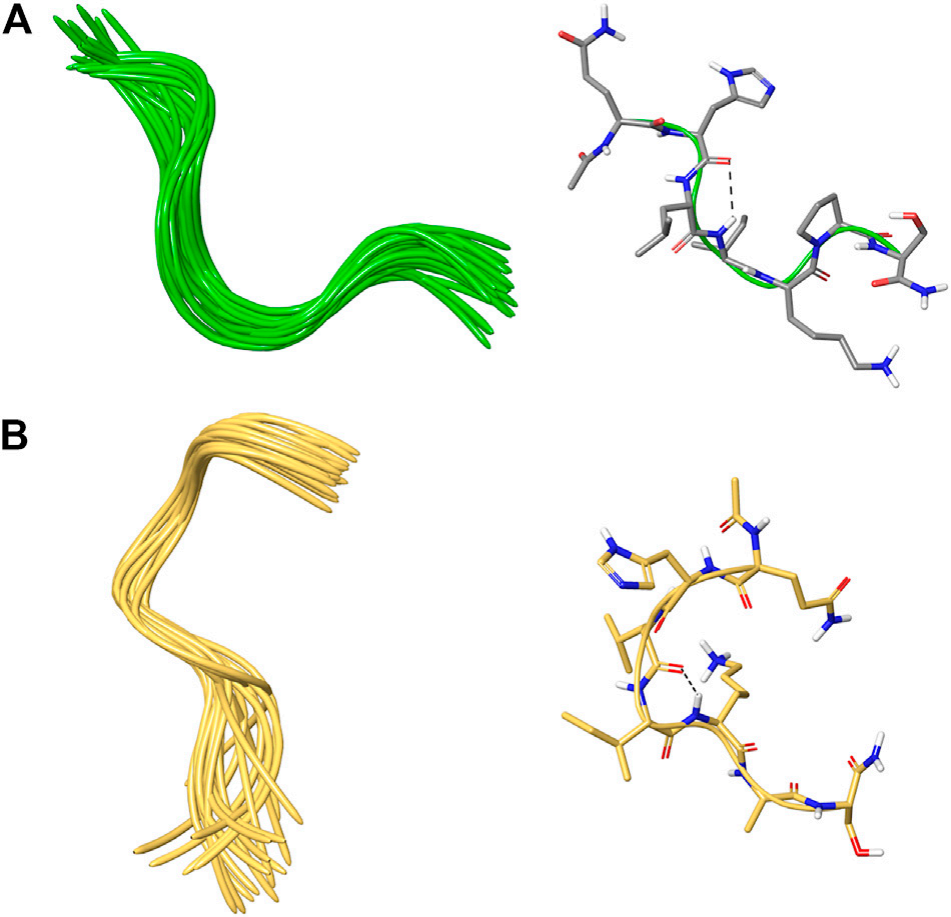
**(A,B)** On the left, superposition of backbone atoms of 20 NMR structures of **1** (green ribbons) and **3** (faded orange ribbons) generated by using CYANA 2.1. **(A,B)** On the right, the average NMR derived structures of **1** and **3**. The atoms are depicted in tube and colored by atom types C, grey for **1**; C, faded orange for **3**; O, red; N, blue; polar hydrogen, white. The dashed lines indicate intramolecular H-bonds responsible of the global fold.

The NMR solution-state structure (HFA/H_2_O) of **1** and **3** were solved following standard procedures (Wüthrich, 1986; Scala et al., 2017; Maulucci et al., 2007), through the ^1^H chemical shift assignments (Tables 3, 4) and collecting inter-proton distance restraints from 2D-NOESY experiments (Figures 3–6). The NMR structure bundle (Figure 7, left) of **1** shows high structural agreement with RMSD of 0.89 Å referenced to the backbone atoms. By means of PROMOTIF software (Hutchinson and Thornton, 1996), the quantitative analysis of ϕ and ψ dihedral angles of the representative structures of **1** was carried out, highlighting a global turn conformation. In detail, the NMR structure backbone analysis of **1** (Figure 7A), revealed the presence of a β-turn (type IV) involving the residues: Leu3–Pro6 (Table 5). Moreover, the observation of intense NOE between H^α^ of Lys5 and H^δ^ of Pro6 revealed the *trans* orientation of the peptide bond connecting these two residues (Wüthrich, 1986; Di Micco et al., 2008; Terracciano et al., 2010). The NMR conformation bundle (Figure 7, left) of **3** presents high structural definition with an RMSD of 0.94 Å referenced to the backbone atoms (Figure 7). As observed for **1**, the quantitative analysis of ϕ and ψ dihedral angles of the representative structures of **3** revealed a global turn arrangement. In particular, the heptapeptide **3** contains an inverse γ-turn (type B) involving the residues Leu3-Lys5 (Table 6). Thus, the residue switch from Pro to Ala in **3** induced to γ-turn conformational arrangement. Moreover, the C-terminal moiety of **3** results are more flexible than in **1**.

**TABLE 3:** ^1^H chemical shifts (ppm) of **1** in HFA/H_2_O (600 MHz, 300 K).

Residue	NH	αH	βH	γH	δH	εH	Others
Gln1	7.62	3.91	1.64	2.03	-	-	-
			1.72				
His2	7.96	4.38	2.86 2.96		6.93	-	-
Leu3	7.61	4.06	1.32	1.20	0.57	-	-
					0.62		
Ile4	7.45	4.08		1.29	0.57	0.62	
				1.35			
Lys5	7.44	4.38	1.55	1.15	1.42	2.72	-
Pro6	-	4.15	1.77	1.69	3.34	-	-
			2.01		3.48		
Ser7	7.57	4.15	3.60		-	-	-
			3.62				

**TABLE 4:** ^1^H chemical shifts (ppm) of **3** in HFA/H_2_O (600 MHz, 300 K).

Residue	NH	αH	βH	γH	δH	εH	Others
Gln1	7.63	3.81	1.61	2.00	-	-	-
His2	7.96	4.23	2.82	-	6.86	-	-
			2.89				
Leu3	7.47	3.95	1.30	1.14	0.51	-	-
					0.56		
Ile4	7.42	3.67	1.51	0.55	0.50	-	
Lys5	7.55	3.93	1.53	1.35	1.44	-	-
Ala6	7.55	3.96	1.09		-	-	-
Ser7	7.52	4.05	3.59		-	-	-

**TABLE 5:** Mean values of φ, ψ, and χ1 angles and αC distances relative to the most representative conformers of **1**.

Peptide	Sequence	i + 1			i + 2			αC distance (Å)
		φ	ψ	χ1	Φ	ψ	χ1	i to i + 3
1	L3-P6	−130.9	29.0	26.9	95	69.4	−147.8	6.7

**TABLE 6:** Mean values of φ, ψ, and χ1 angles and αC distances relative to the most representative conformers of **3**.

Peptide	Sequence	i + 1			αC distance (Å)
		φ	Ψ	χ1	i to i + 2
**3**	L3-K5	−85.3	51.4	−44.8	5.5

### Biological Assays

**FIGURE 8:**
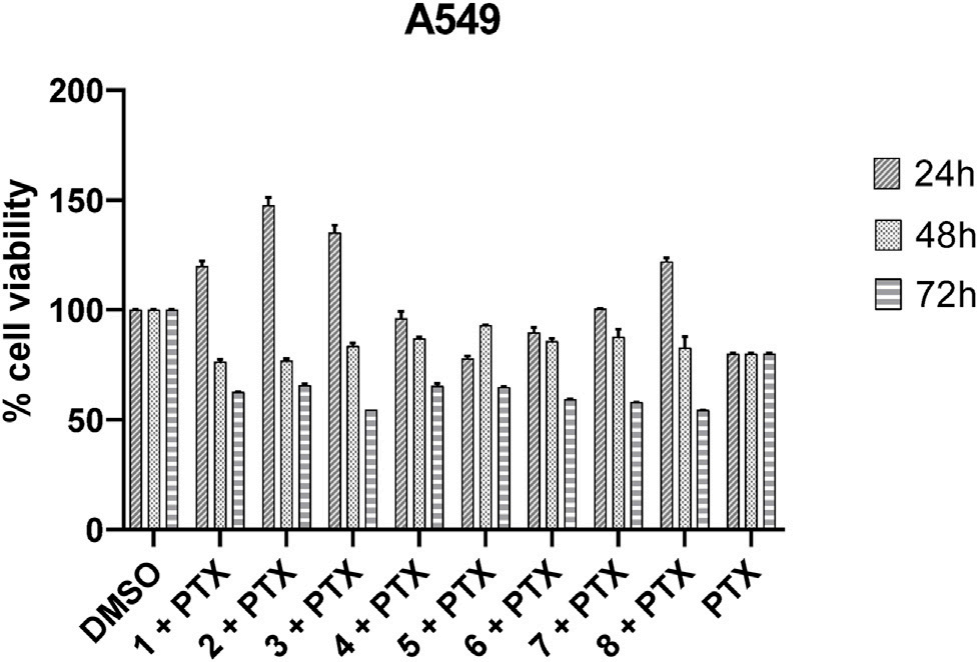
Inhibition of A549 cell viability in cells treated with paclitaxel (800 nM) and peptides **1**–**8** (100 μM). Cells were plated in triplicate in 384-well plates and treated with PTX and peptides (DMSO was used as a positive control) for 24, 48, or 72 h. Cell viability was measured using CellTiter assay as described in the Materials and Methods section. Cell viability was expressed as a percentage of control and each column represents the mean ± SD of three independent experiments.

Cell viability was measured on A549 cell lines treated with a single administration of 10, 50, 100, and 150 μM peptides at 24, 48, and 72 h. After treatment with peptides **1**–**8**, the A549 lung cancer cells did not show significantly reduced viability up to 150 μM. The same results were obtained by treating different cell lines, such as: MCF7, PC3, M14, and Hela. Then, the synergistic effects of paclitaxel and peptides **1**–**8** in A549 cells were investigated. As shown in Figure 8, the replacement of the C-terminal polar residue, Ser7, with an alanine basically preserves the biological activity at 72 h of the corresponding analogues **2** compared to reference peptide **1**. The substitution of positively charged residue, Lys5, and of the hydrophobic residue, Ile4, with Ala in the analogues **4** and **5** determines a similar biological profile of **2**. In derivative **6** and **7**, the substitution of the amino acids Leu3 and His2 slightly improves the paclitaxel cytotoxicity. Finally, the change of the constrained conformational residue, Pro6, and of the polar residue, Gln1, in the corresponding analogue **3** and **8** determines a decrease of cell viability compared to reference peptide **1**. In fact, ∼50% cell growth inhibition was observed in cells treated with **3** + PTX and **8** + PTX, with a time-dependent manner.

## Discussion

FHIT-mimetic peptide (7–13, QHLIKPS) has been reported to interact with ANXA4 restoring chemosensitivity to paclitaxel in lung cancer cells. In this study, the systematic use of an alanine scan was used to delineate the contribution of individual amino acids to FHIT (7–13)-ANXA4 interaction. This interaction, which was originally established by Gaudio et al., has never been quantified in terms of affinity measurements (K_D_). Thus, we decided to afford a structure-activity relationship study by means of a multidisciplinary approach allowing the correlation of peptide chemical modifications and pharmacological properties for a rational design approach. Biophysical binding experiments, which measure physical property changes upon the direct ligand binding, generally serve as the basis for the initial screening. To address the limitations of each method, we decided to pursue a multi-pronged screening strategy, by investigating the putative binding of peptides toward ANXA4 through three complementary techniques: MST, SPR, and nanoDSF. Both SPR and MST demonstrated the binding of **1**–**8** to ANX4, showing an agreement in the affinity profiles of peptides. In detail, the peptides **3** and **4** (Table 1) showed the highest affinity toward ANXA4, followed by **2**. The remaining peptides presented comparable values ranging from 1.13 to 6.4 μM in MST, and from 0.84 to 1.52 μM in SPR. Compared to SPR and MST, the nanoDSF assay did not give us relevant information about the binding between protein and peptides. In fact, all ligands showed similar ΔTm values not useful for a structure-activity relationship investigation.

Successively, a conformational analysis was performed by CD and NMR spectroscopy. CD studies revealed that peptide **1** and more specifically peptide **3** assume a better-defined conformation than all synthetized analogues. This was later confirmed by more detailed NMR studies on peptide **1** and **3**, revealing global turn conformations for both peptides. In fact, the backbone inspection of 7–13 fragment, extracted from X-ray structure of FHIT protein (PDB ID: 4FIT) (Lima et al., 1997), revealed that Leu9 and Ile10 constitute a β-strand, whereas the N- and C-terminal residues are involved in specific spatial arrangements with extra fragment amino acids. In detail, Gln7 and His8 are involved in β-turn (type IV) with Phe5 and Gly6, whereas Pro12 and Ser13 fold in a G helix with Val14. These structural observations are in line with global turn conformations observed for **1** and **3** involving Leu3 and Ile4, suggesting these residues as structural requirements for the resulting peptide activity as shown by MST and SPR binding experiments (Table 2). These data are also in agreement with the biological results, especially for peptide **3**, in which the replacement of the proline with alanine improve the biological activity. These findings suggest that the activity of the peptide is given by the conformation adopted rather than by amino acidic sequence.

In conclusion, our major goal was to define a multidisciplinary biophysical and structural screening study allowing the identification of the structural determinants responsible for activity. This approach represents a valuable starting point for the future development of synthetic FHIT-mimetic small molecules that, combined with drugs already available on the market, would be able to overcome chemoresistance.

## Data Availability

The original contributions presented in the study are included in the article/Supplementary Material. Further inquiries can be directed to the corresponding author.
