# Corrigendum: Overcome Chemoresistance: Biophysical and Structural Analysis of Synthetic FHIT-Derived Peptides

**DOI:** 10.3389/fmolb.2022.915838

**Published:** 2022-04-29

**Authors:** Maria Carmina Scala, Simone Di Micco, Delia Lanzillotta, Simona Musella, Veronica Di Sarno, Barbara Parrino, Stella Maria Cascioferro, Giuseppe Bifulco, Francesco Trapasso, Pietro Campiglia, Marina Sala

**Affiliations:** ^1^ Department of Pharmacy, University of Salerno, Fisciano, Italy; ^2^ European Biomedical Research Institute of Salerno (EBRIS), Salerno, Italy; ^3^ Department of Experimental and Clinical Medicine, University Magna Græcia, Campus S. Venuta, Catanzaro, Italy; ^4^ Department of Biological Chemical and Pharmaceutical Sciences and Technologies (STEBICEF), University of Palermo, Palermo, Italy

**Keywords:** chemoresistance, peptide, FHIT, annexin A4, biophysical assay

An author name was incorrectly spelled as “Stella Maria Casciofierro”. The correct spelling is “Stella Maria Cascioferro”.

The authors apologize for this error and state that this does not change the scientific conclusions of the article in any way. The original article has been updated.

