# Supplementary material for: Overcome Chemoresistance: Biophysical and Structural Analysis of Synthetic FHIT-Derived Peptides
Source: Front Mol Biosci. 2021 Nov 25;8:715263. doi: 10.3389/fmolb.2021.715263 (PMC8655160; doi:10.3389/fmolb.2021.715263)
Supplement: Supplementary file 1 [file DataSheet1.PDF]

Supplementary Material

Overcome chemoresistance: biophysical and structural analysis of synthetic FHIT-derived peptides

Maria Carmina Scala [a], Simone Di Micco [b], Delia Lanzillotta[c], Simona Musella[b], Veronica Di Sarno [a], Barbara Parrino [d], Stella Casciofierro [d], Giuseppe Bifulco[a], Francesco Trapasso[c], Pietro Campiglia[a] and Marina Sala[a*]

[a]*Department of Pharmacy, University of Salerno, Via Giovanni Paolo II 132, 84084, Fisciano, Italy;*
[b] *European Biomedical Research Institute of Salerno (EBRIS), Via S. de Renzi 50, 84125 Salerno (SA). Italy;*
[c]*Department of Experimental and Clinical Medicine, University Magna Græcia, Campus S. Venuta, via Europa 88100, Catanzaro, Italy;*
[d]*Department of Biological Chemical and Pharmaceutical Sciences and Technologies (STEBICEF), University of Palermo, I-90128 Palermo, Italy.*

*** Correspondence:**

Marina Sala

msala@unisa.it

Supplementary schemes and figures:

**Table S1:**	Analytical data of peptides **1-8**	S2
**Figure S1-S8:**	HRMS spectra and HPLC chromatograms of peptides **1-8**	S2-S9
**Figure S9:**	CD Spectra of peptides **1-8**	S10
**Figure S10-12:**	MST binding curves of peptides **2, 5-8**	S10-S11
**Figure S13-20:**	Sensorgrams of peptides **1-8**	S11-S15
**Figure S21:**	Ramachandran plot of NMR derived bundle of **1**	S16
**Figure S22:**	Ramachandran plot of NMR derived bundle of **3**	S17
**References**	....	S18

Table S1. Analytical data of peptides **1-8**.

PEPTIDE	NAME	SEQUENCE	HPLC *k'*[a]	ESI-MS
**FHIT 7-13**	**1**	QHLIKPS	4.87	863.3271
**S7A**	**2**	QHLIKPA	4.66	848.4563
**P6A**	**3**	QHLIKAS	3.88	837.9574
**K5A**	**4**	QHLIAPS	2.30	806.4734
**I4A**	**5**	QHLAKPS	3.66	821.4092
**L3A**	**6**	QHAIKPS	1,72	821.3272
**H2A**	**7**	QALIKPS	5.00	797.3838
**Q1A**	**8**	AHLIKPS	4.82	806.4001

[a] k'=[(peptide retention time-solvent retention time)/solvent retention time].

Supplementary figures of Mass spectrometry and HPLC of peptides used in the study

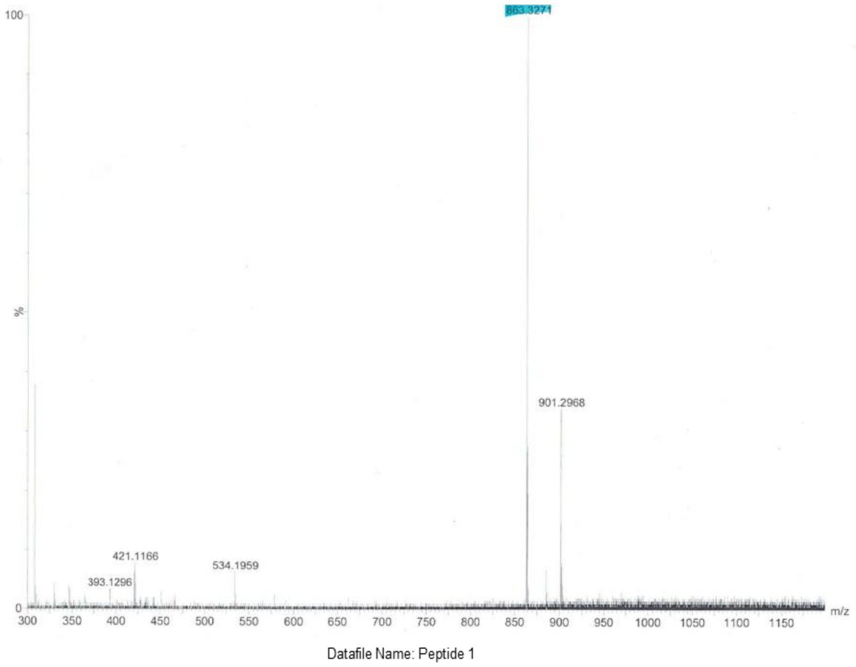

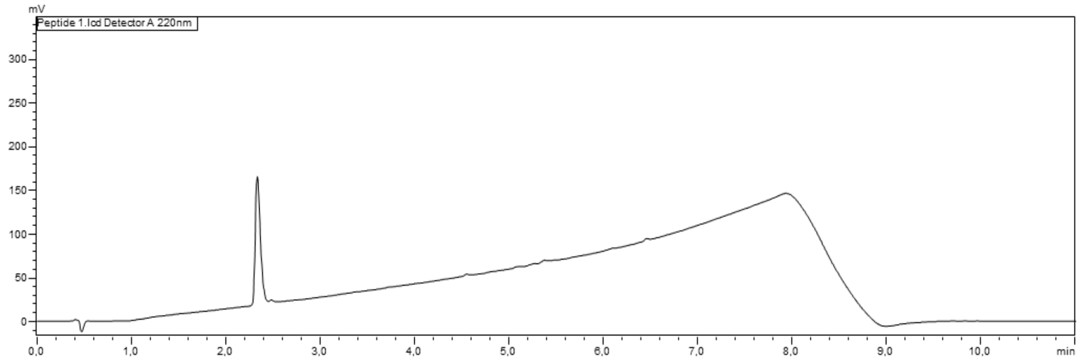

Figure S1. HR-ESI-MS of Peptide **1** ion $[M+H]^+$ and analytical HPLC trace at 220 nm.

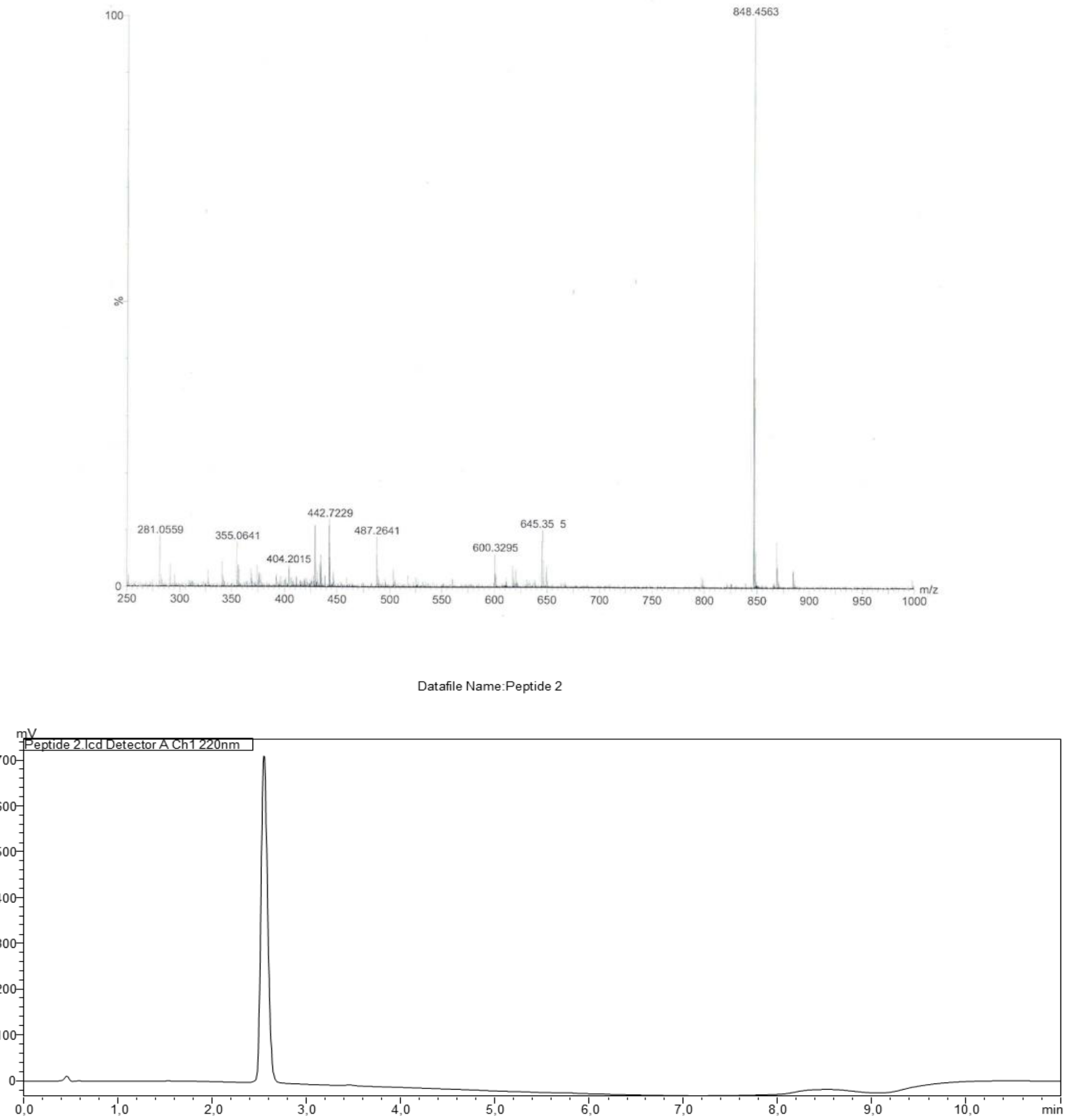

Figure S2. HR-ESI-MS of Peptide **2** ion $[M+H]^+$ and analytical HPLC trace at 220 nm.

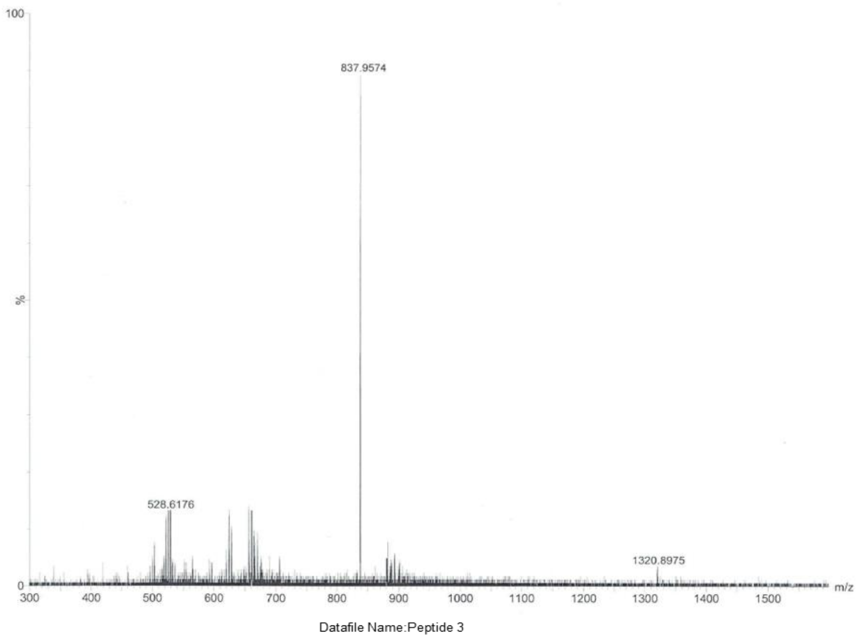

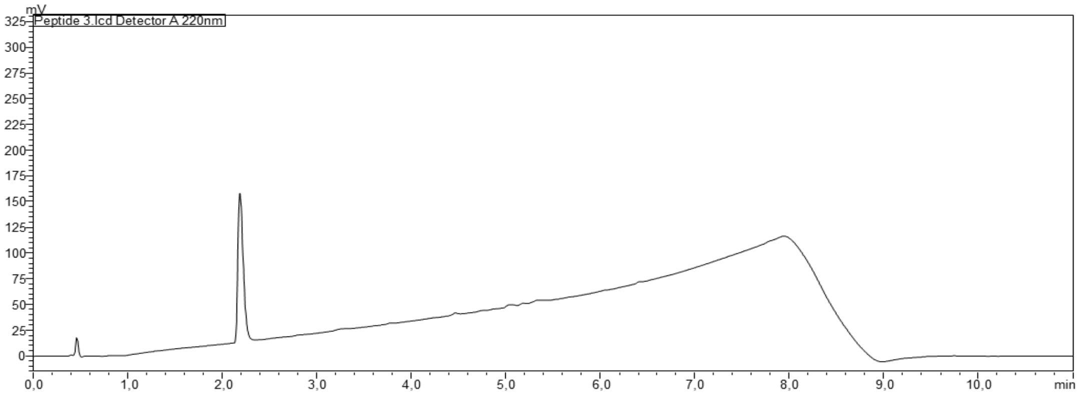

Figure S3. HR-ESI-MS of Peptide **3** ion $[M+H]^+$ and analytical HPLC trace at 220 nm.

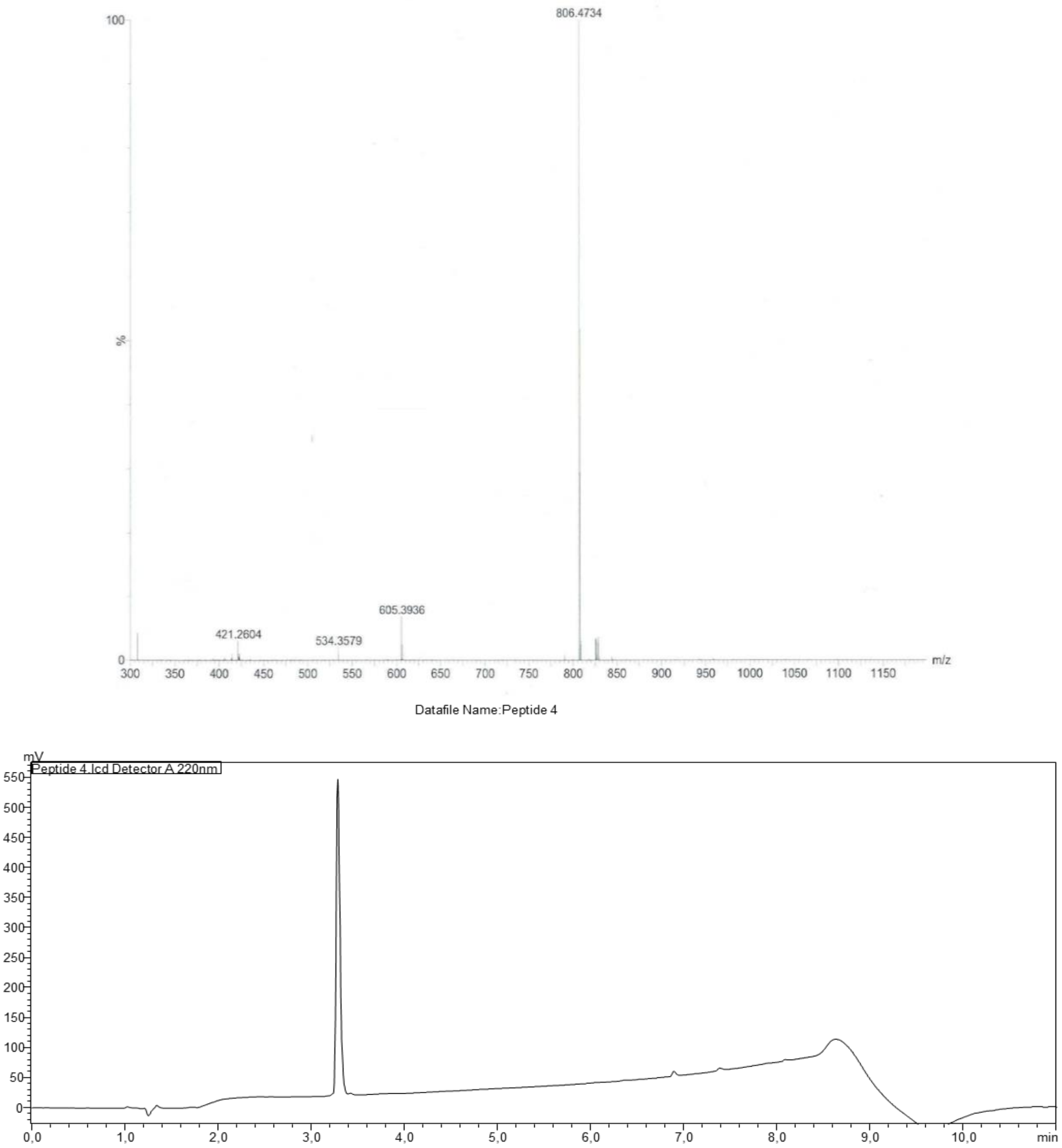

Figure S4. HR-ESI-MS of Peptide **4** ion [M+H]$^+$ and analytical HPLC trace at 220 nm.

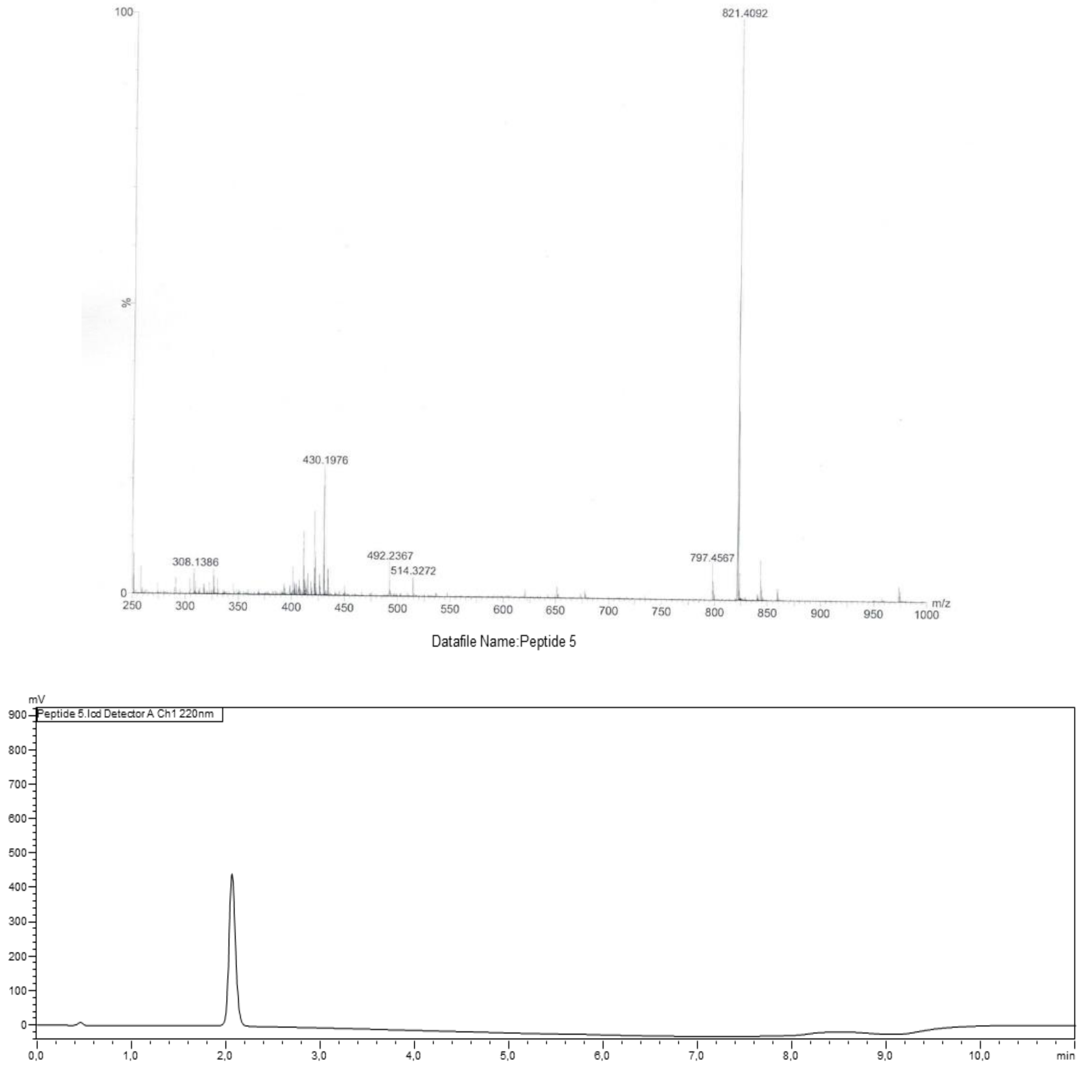

Figure S5. HR-ESI-MS of Peptide **5** ion $[M+H]^+$ and analytical HPLC trace at 220 nm.

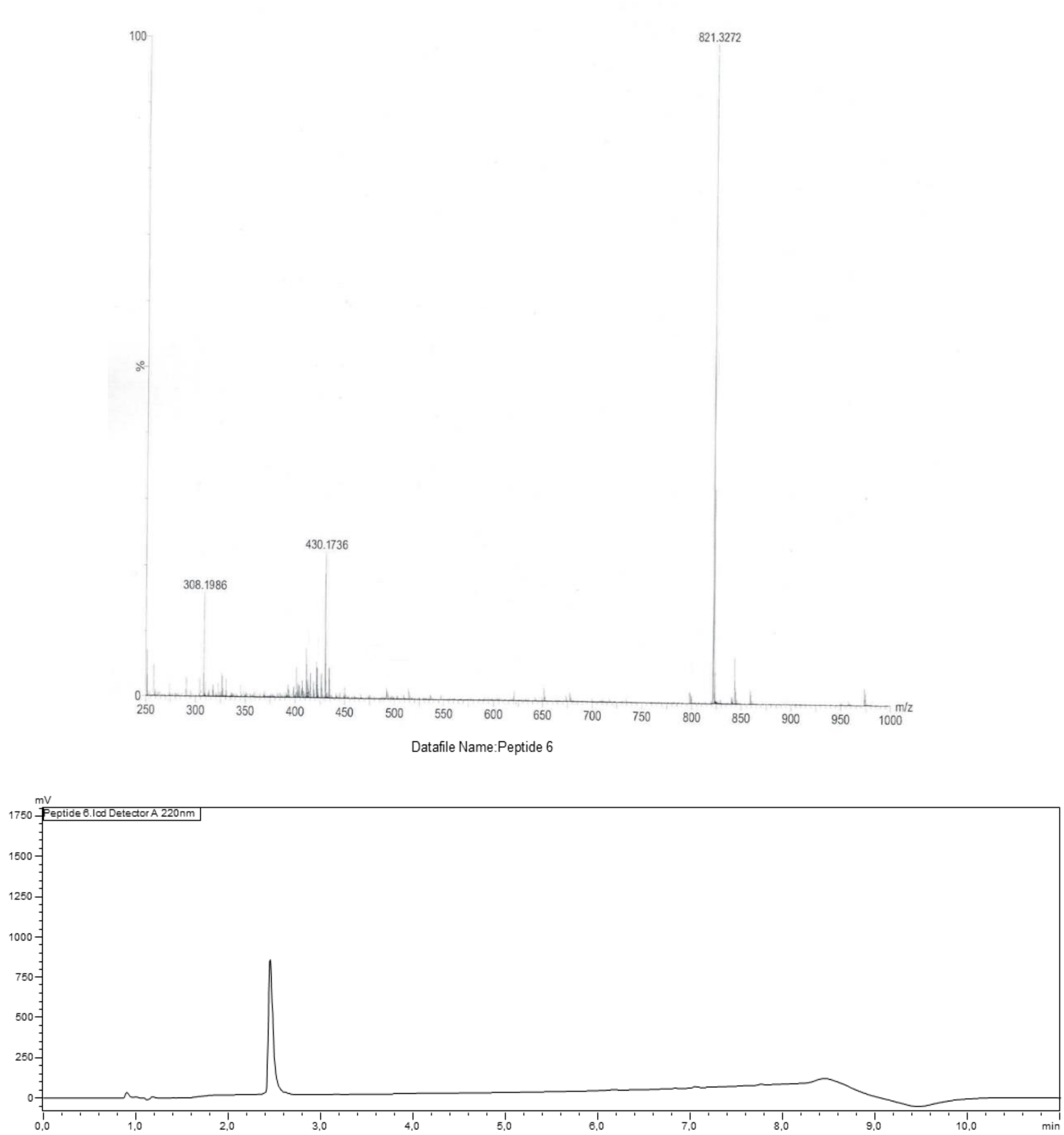

Figure S6. HR-ESI-MS of Peptide **6** ion $[M+H]^+$ and analytical HPLC trace at 220 nm.

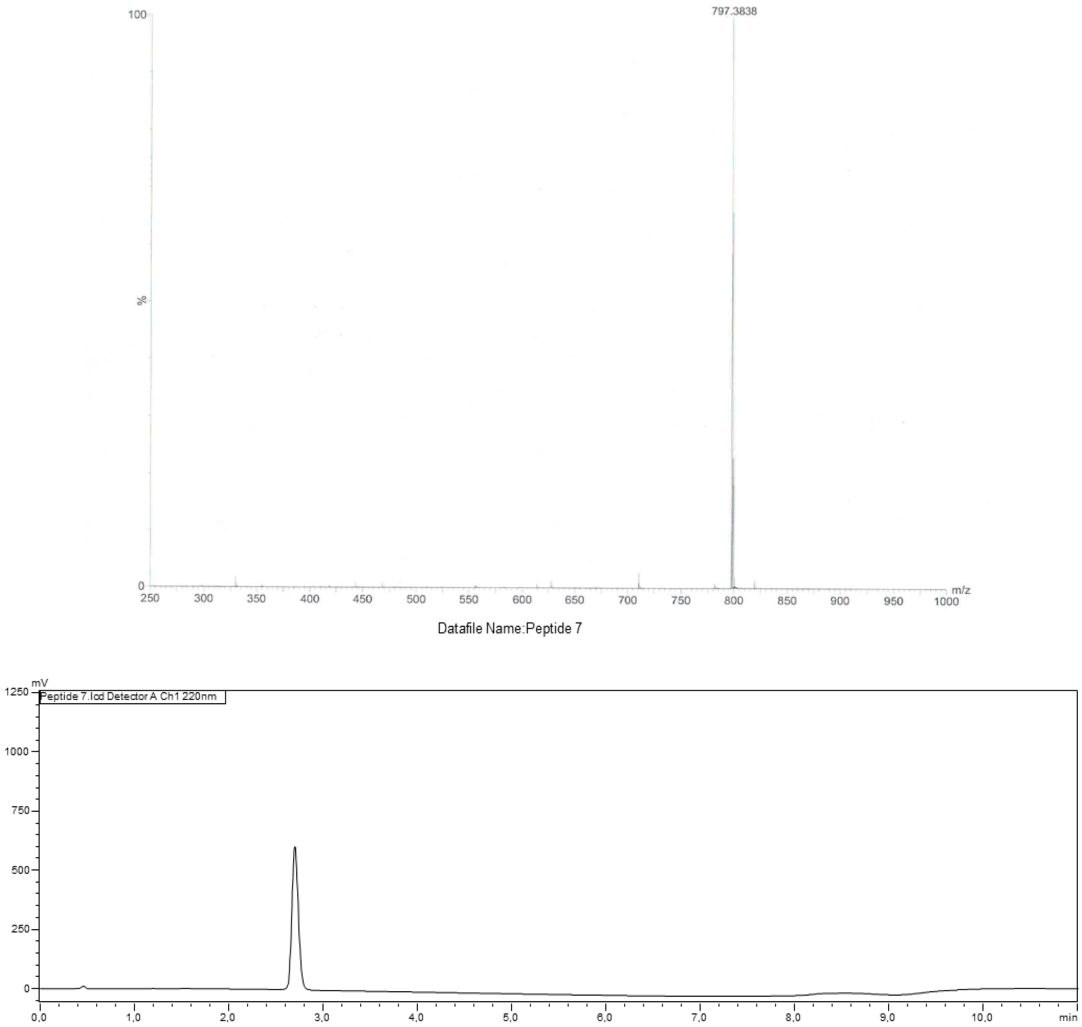

Figure S7. HR-ESI-MS of Peptide **7** ion $[M+H]^+$ and analytical HPLC trace at 220 nm.

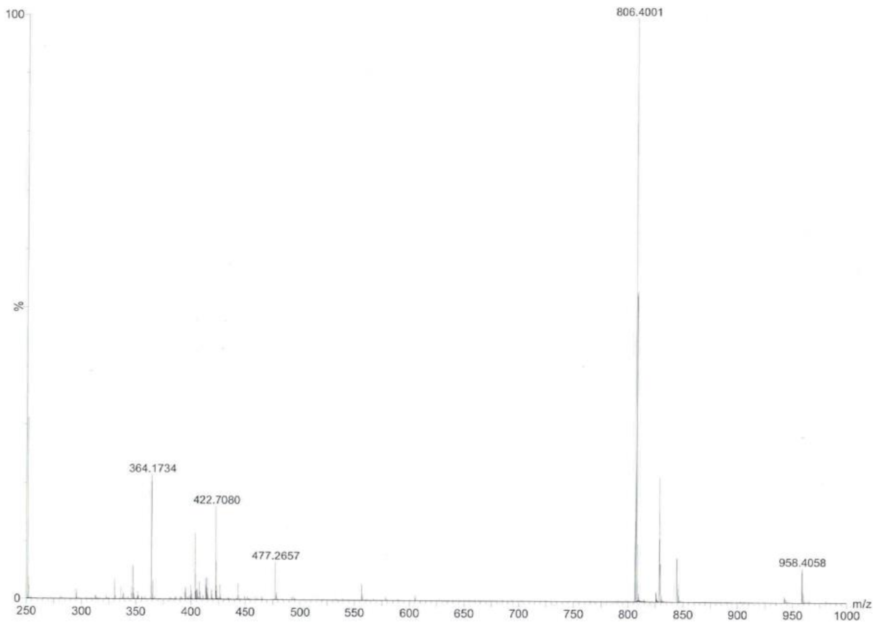

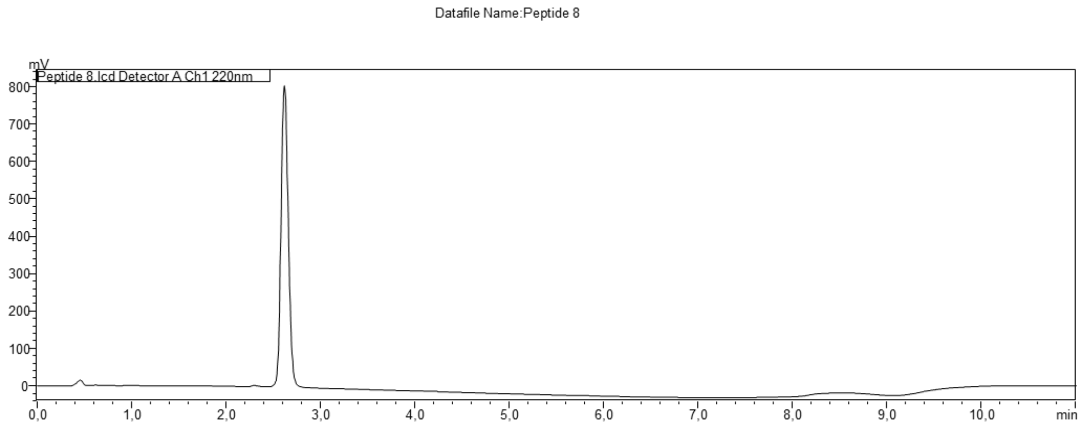

Figure S8. HR-ESI-MS of Peptide **8** ion $[M+H]^+$ and analytical HPLC trace at 220 nm.

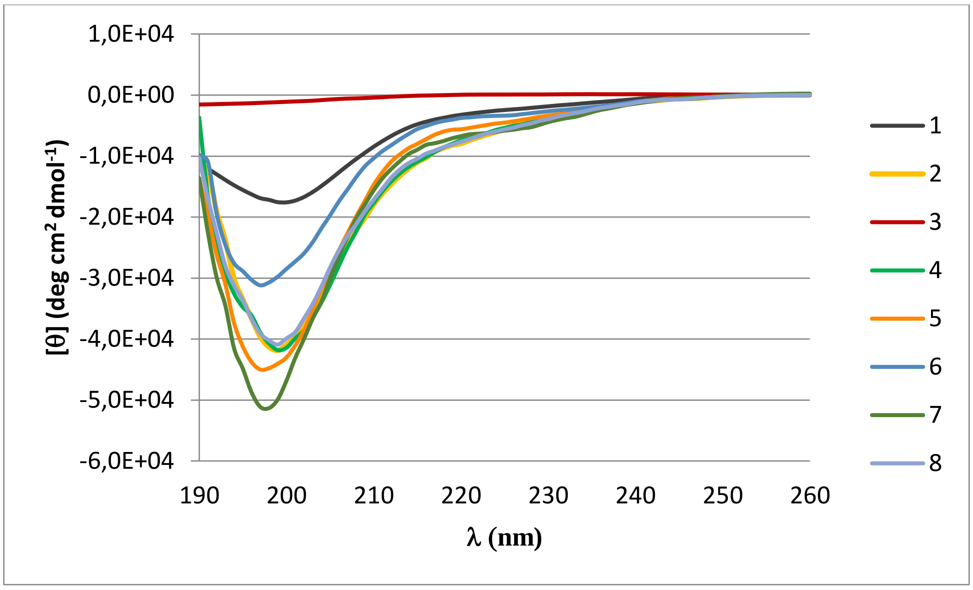

Figure S9. CD Spectra of selected peptides in PBS.

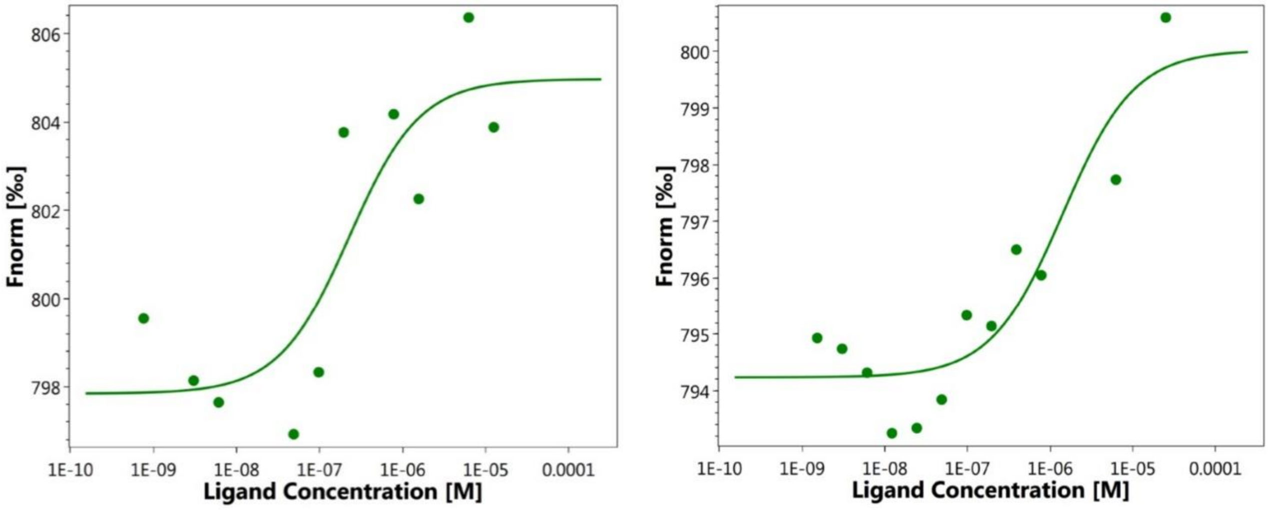

Figure S10. MST binding curve of peptide **2-5** to ANX A4.

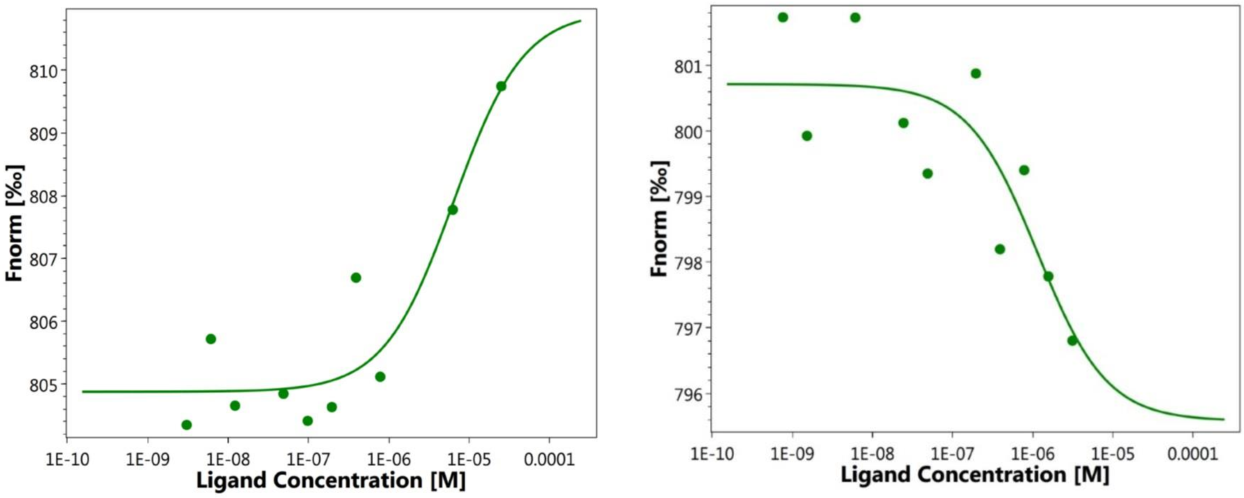

Figure S11. MST binding curve of peptide **6-7** to ANX A4.

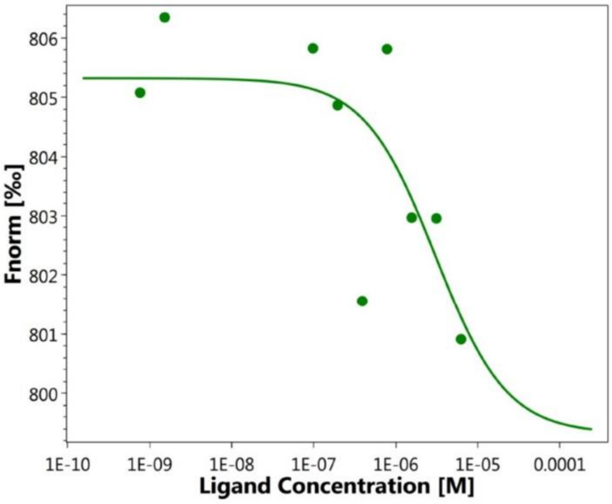

Figure S12. MST binding curve of peptide **8** to ANX A4.

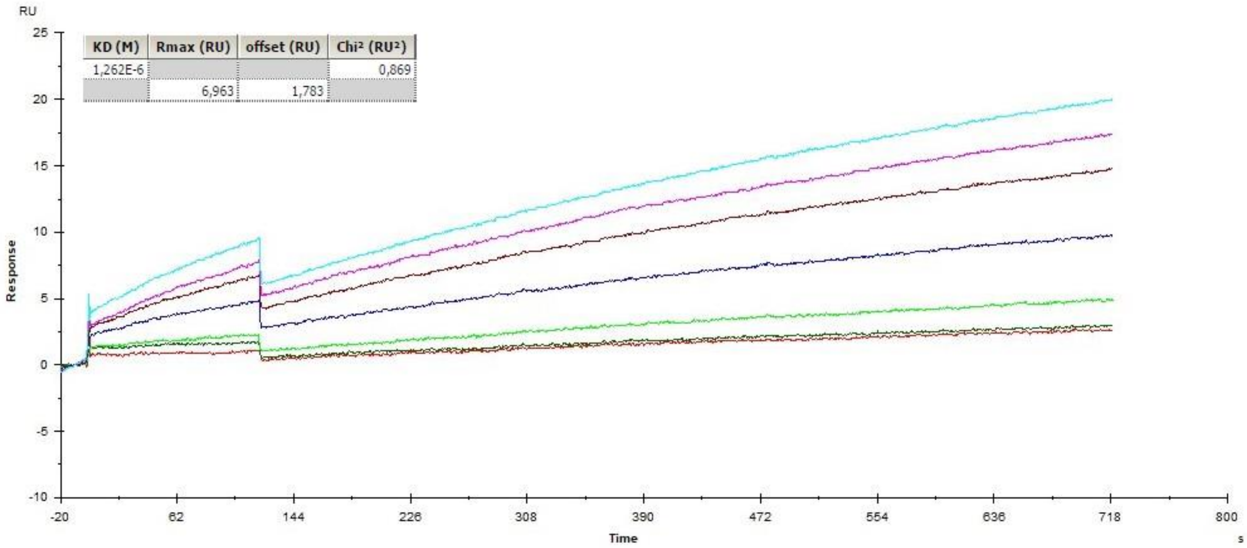

Figure S13. SPR sensorgrams between ANX A4 (immobilized on the sensor chip surface) and the peptide **1**.

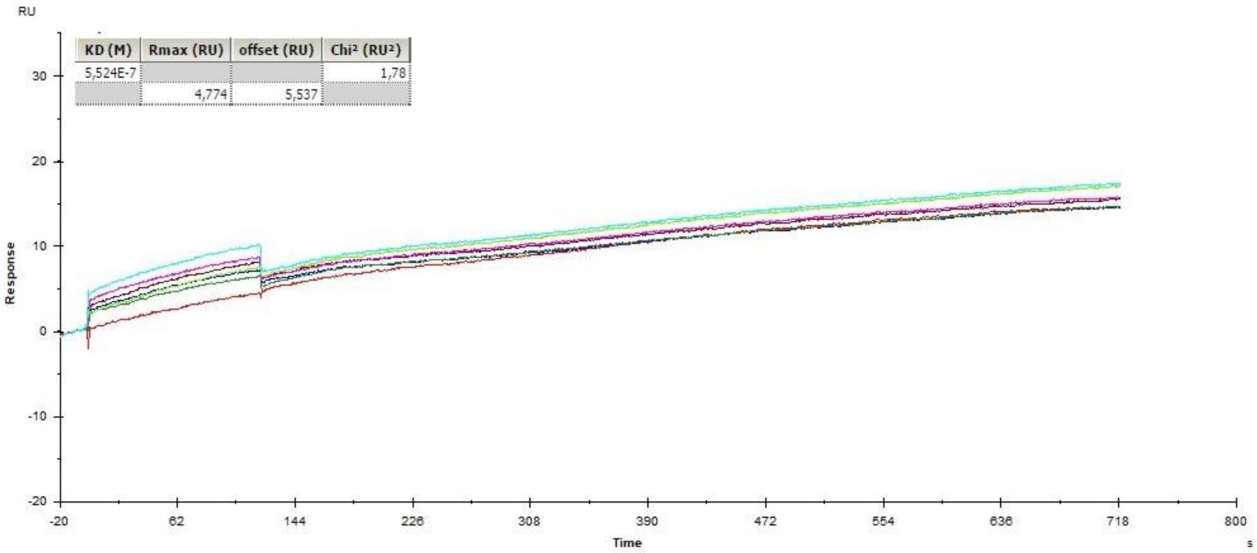

Figure S14. SPR sensorgrams between ANX A4 (immobilized on the sensor chip surface) and the peptide **2**.

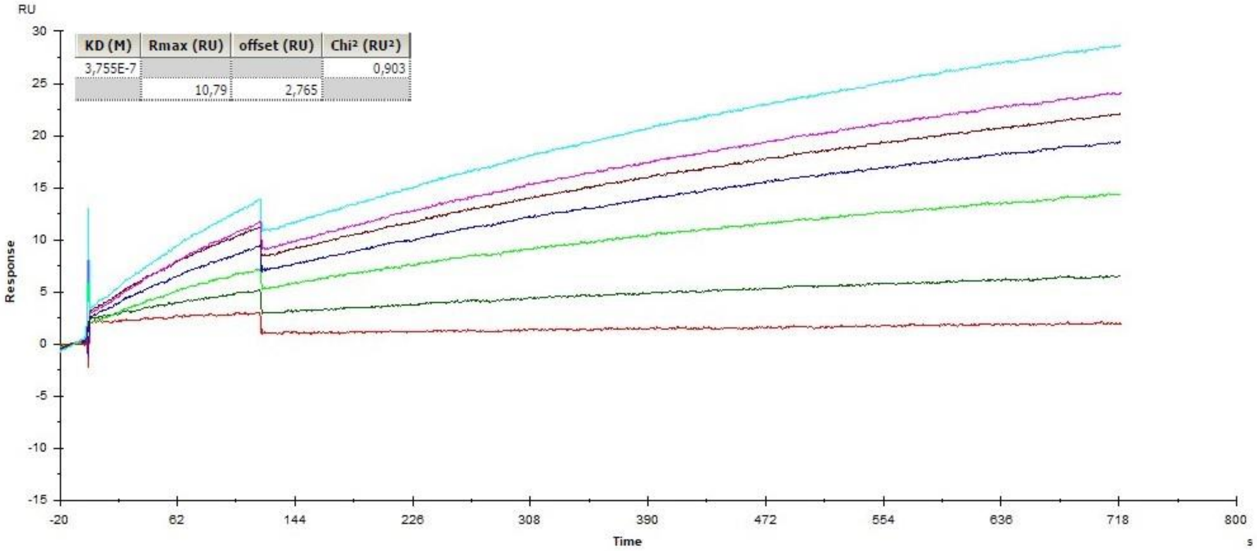

Figure S15. SPR sensorgrams between ANX A4 (immobilized on the sensor chip surface) and the peptide **3**.

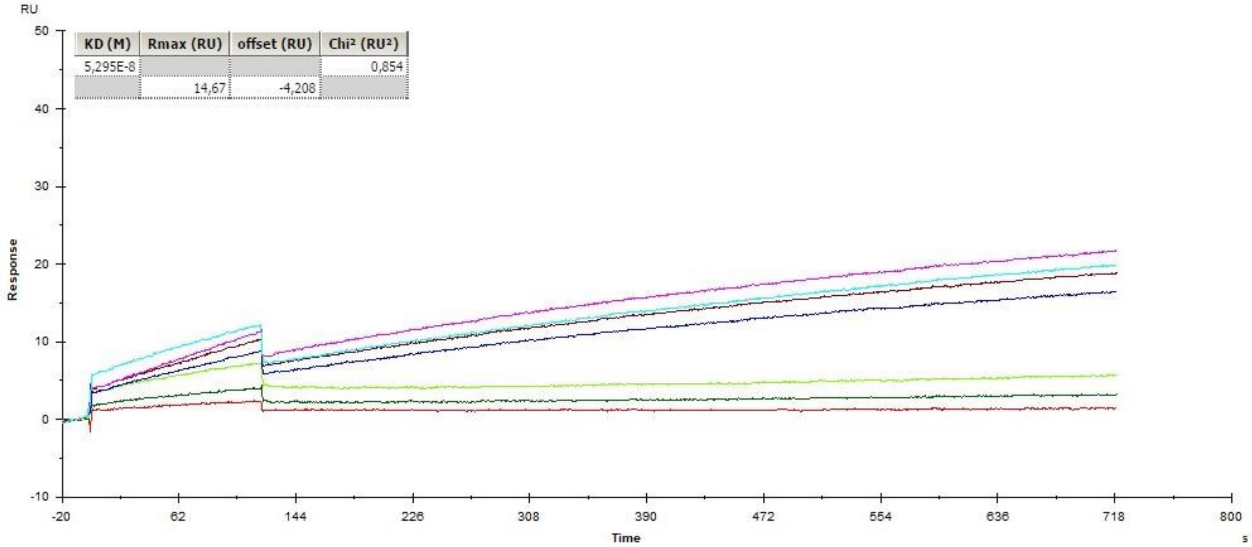

Figure S16. SPR sensorgrams between ANX A4 (immobilized on the sensor chip surface) and the peptide **4**.

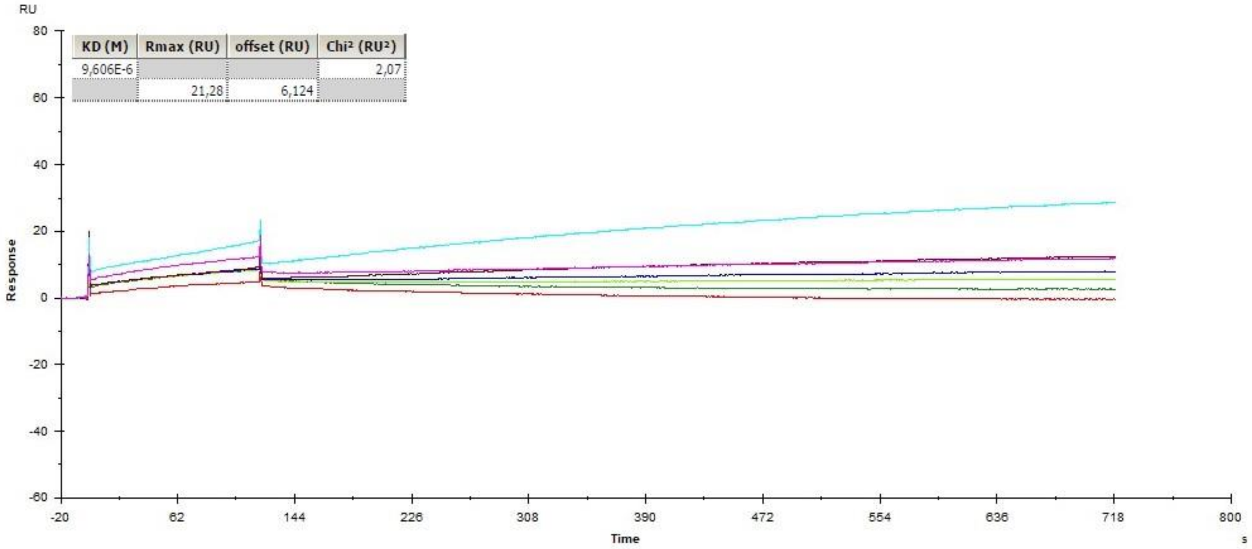

Figure S17. SPR sensorgrams between ANX A4 (immobilized on the sensor chip surface) and the compound **5**.

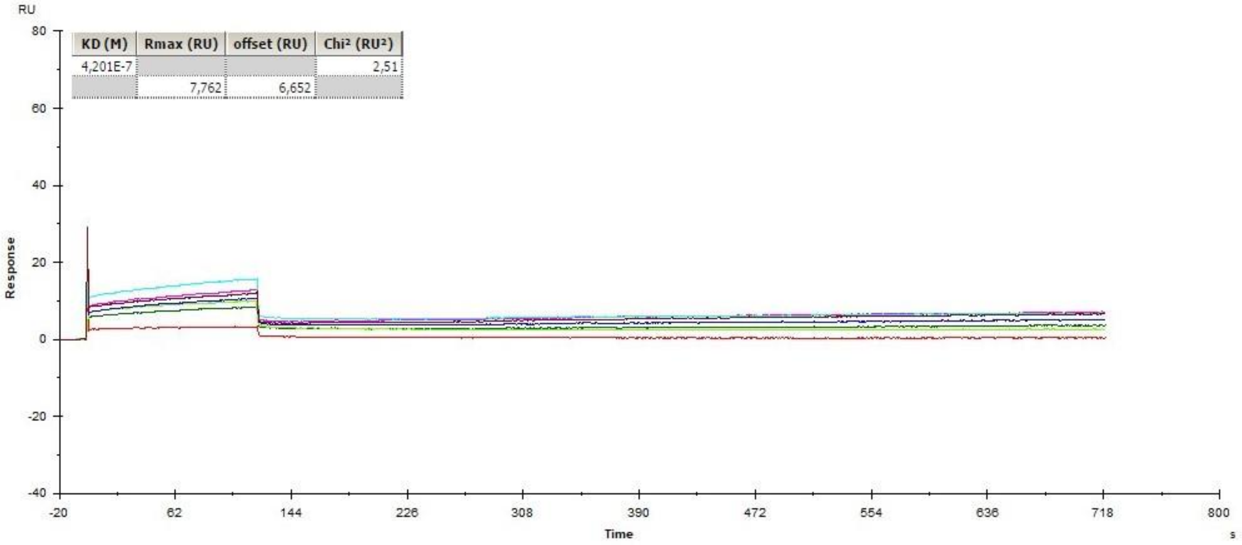

Figure S18. SPR sensorgrams between ANX A4 (immobilized on the sensor chip surface) and the compound **6**.

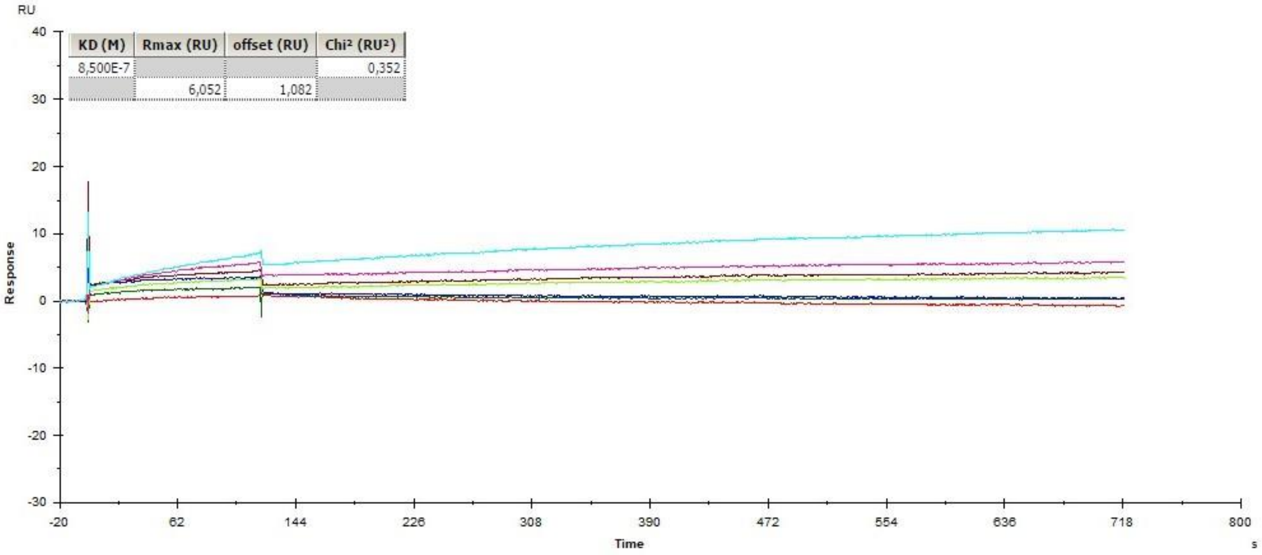

Figure S19. SPR sensorgrams between ANX A4 (immobilized on the sensor chip surface) and the compound **7**.

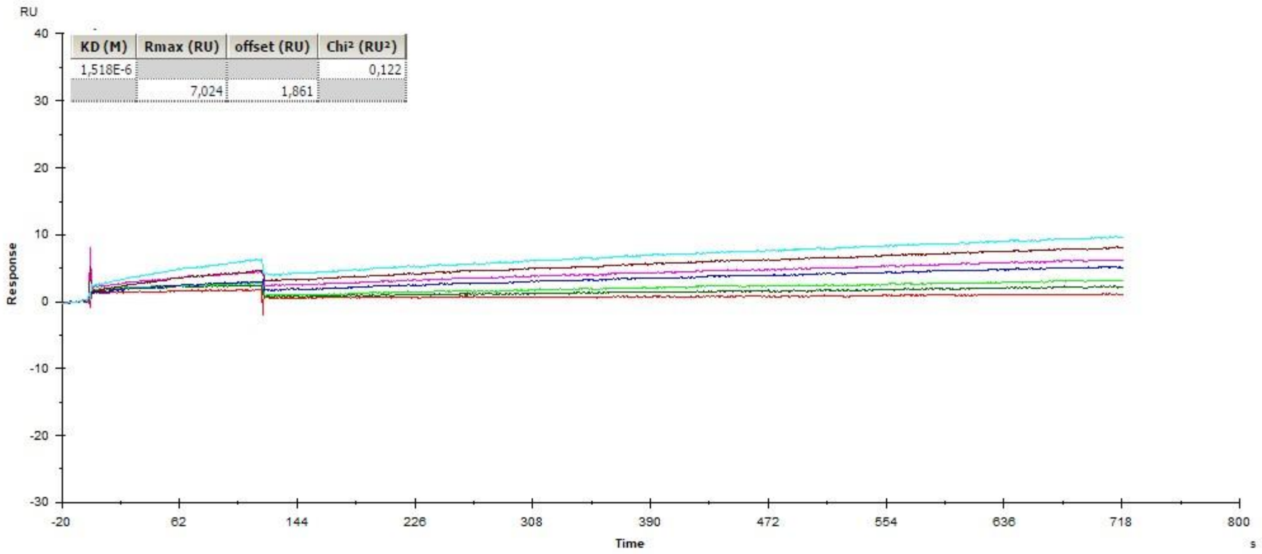

Figure S20. SPR sensorgrams between ANX A4 (immobilized on the sensor chip surface) and the compound **8**.

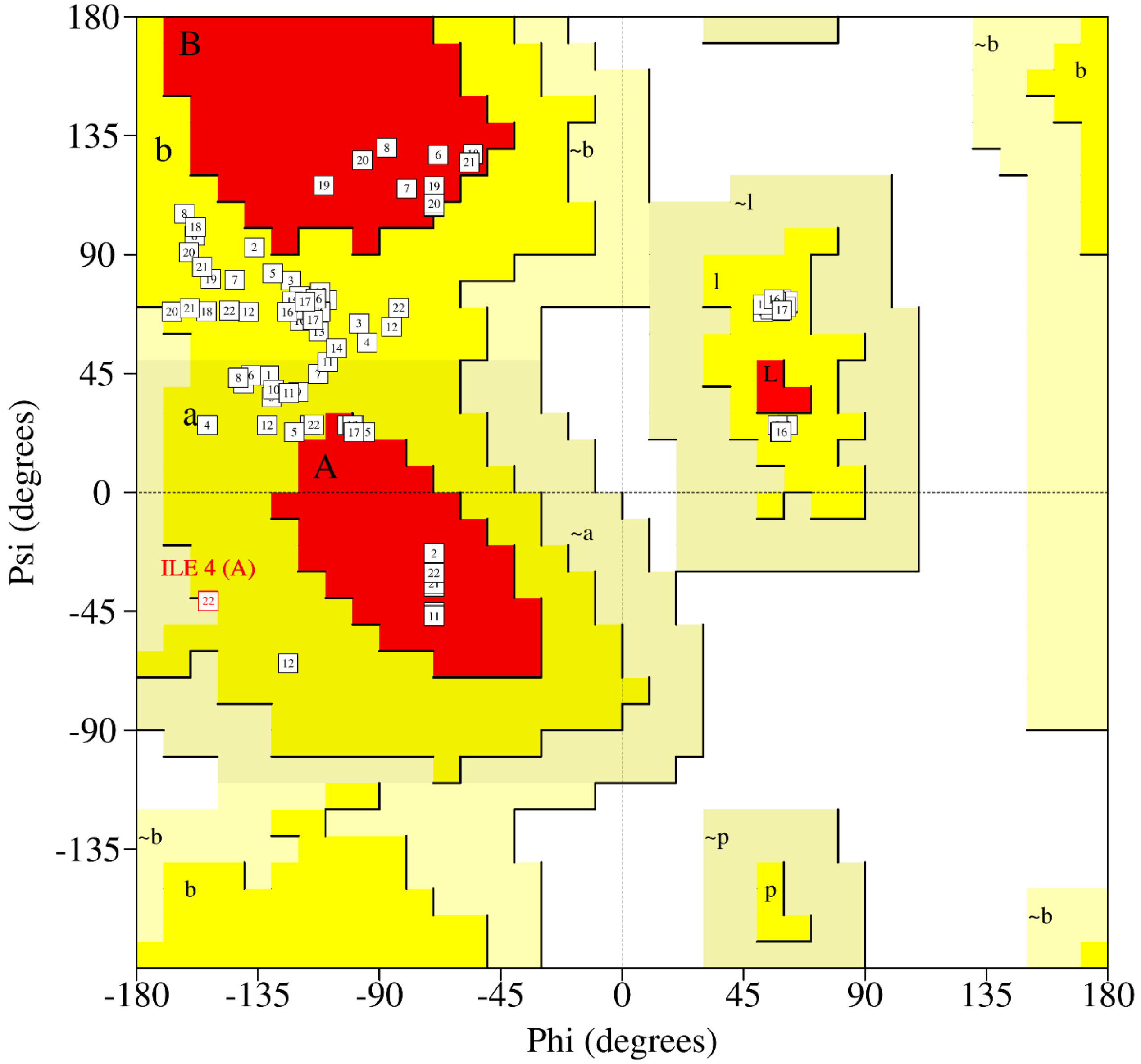

Figure S21. Ramachandran plot of NMR derived bundle of **1**, calculated by PROCHECK[i] software.

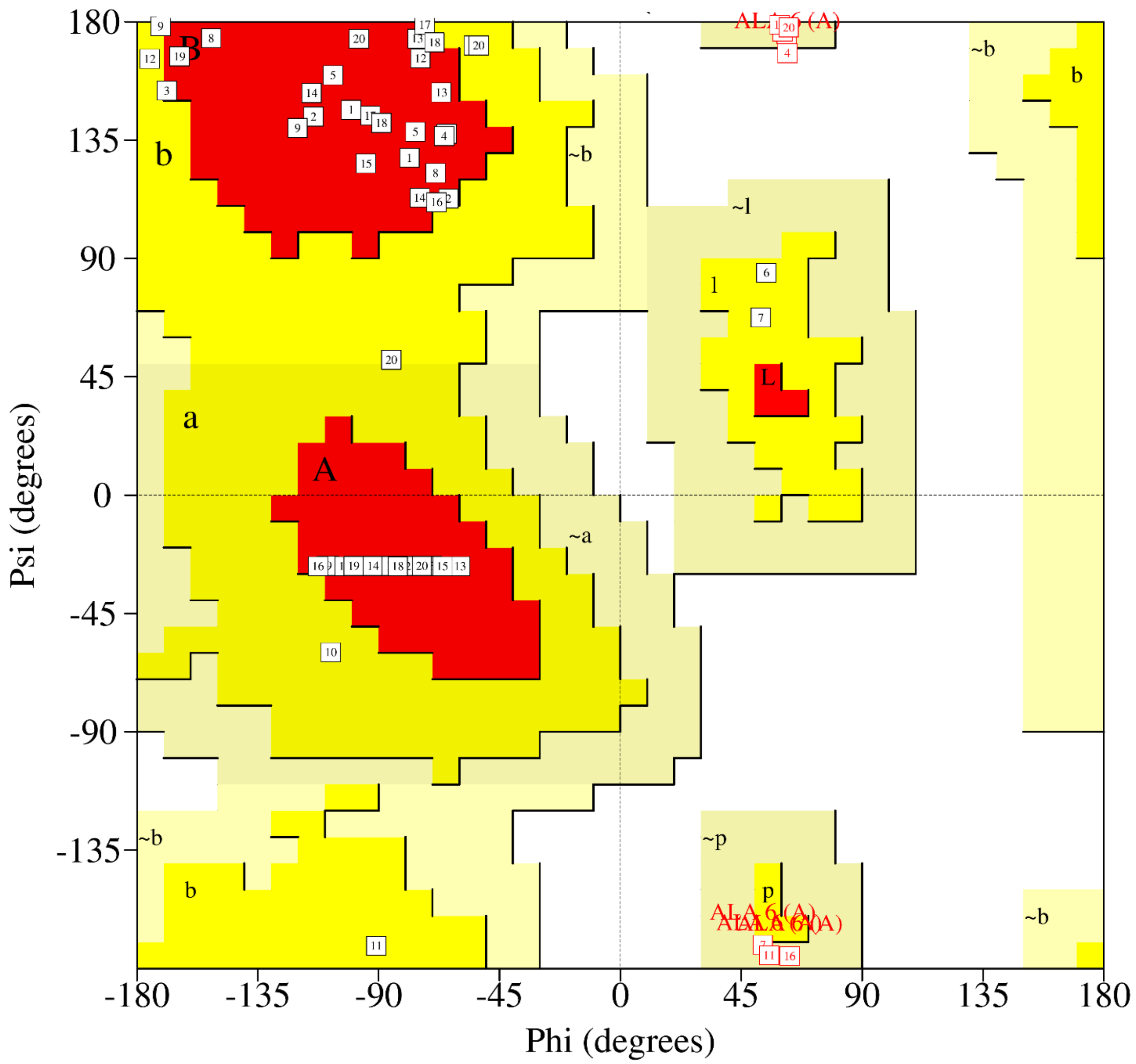

Figure S22. Ramachandran plot of NMR derived bundle of **3**, calculated by PROCHECK[i] software.

References

[i] Laskowski, R. A., Rullmannn, J. A., MacArthur, M. W., Kaptein, R., Thornton, J. M. AQUA and PROCHECK-NMR: programs for checking the quality of protein structures solved by NMR. *J. Biomol. NMR.* **8**, 477-486 (1996).
